# Virus-specific T_RM_ cells of both donor and recipient origin reside in human kidney transplants

**DOI:** 10.1172/jci.insight.172681

**Published:** 2023-11-08

**Authors:** Daphne M. Hullegie-Peelen, Hector Tejeda Mora, Dennis A. Hesselink, Eric M.J. Bindels, Thierry P.P. van den Bosch, Marian C. Clahsen-van Groningen, Marjolein Dieterich, Sebastiaan Heidt, Robert C. Minnee, Georges M.G.M. Verjans, Martin J. Hoogduijn, Carla C. Baan

**Affiliations:** 1 Department of Internal Medicine, Nephrology and Transplantation, Erasmus University Medical Center (Erasmus MC) Transplant Institute,; 2 Department of Haematology, and; 3 Department of Pathology, Erasmus MC Transplant Institute, University Medical Center Rotterdam, Rotterdam, Netherlands.; 4 Institute of Experimental Medicine and Systems Biology, Rheinisch-Westfälische Technische Hochschule (RWTH) Aachen University, Aachen, Germany.; 5 Department of Immunology, Leiden University Medical Center, Leiden, Netherlands.; 6 Department of Surgery, Division of Hepatopancreatobiliary and Transplant Surgery, Erasmus MC Transplant Institute, University Medical Center Rotterdam, Rotterdam, Netherlands.; 7 HerpeslabNL of the Department of Viroscience, University Medical Center Rotterdam, Rotterdam, Netherlands.

**Keywords:** Immunology, Transplantation, Organ transplantation, T cells

## Abstract

Tissue-resident lymphocytes (TRLs) are critical for local protection against viral pathogens in peripheral tissue. However, it is unclear if TRLs perform a similar role in transplanted organs under chronic immunosuppressed conditions. In this study, we aimed to characterize the TRL compartment in human kidney transplant nephrectomies and examine its potential role in antiviral immunity. The TRL compartment of kidney transplants contained diverse innate, innate-like, and adaptive TRL populations expressing the canonical residency markers CD69, CD103, and CD49a. Chimerism of donor and recipient cells was present in 43% of kidney transplants and occurred in all TRL subpopulations. Paired single-cell transcriptome and T cell receptor (TCR) sequencing showed that donor and recipient tissue–resident memory T (T_RM_) cells exhibit striking similarities in their transcriptomic profiles and share numerous TCR clonotypes predicted to target viral pathogens. Virus dextramer staining further confirmed that CD8 T_RM_ cells of both donor and recipient origin express TCRs with specificities against common viruses, including CMV, EBV, BK polyomavirus, and influenza A. Overall, the study results demonstrate that a diverse population of TRLs resides in kidney transplants and offer compelling evidence that T_RM_ cells of both donor and recipient origin reside within this TRL population and may contribute to local protection against viral pathogens.

## Introduction

Tissue-resident lymphocytes (TRLs) are noncirculating lymphocytes found in nonlymphoid tissues, including the kidneys (1–3). Under physiological conditions, TRLs are key players in local immune surveillance, as evidenced by their rapid and strong response against invading pathogens (2, 4–6). However, the characteristics of TRLs and their potential to provide antimicrobial protection in transplanted kidneys have not been fully established.

At the time of procurement, a transplanted kidney contains the donor tissue–resident immune cell compartment, including donor tissue–resident memory T (T_RM_) cells (7–9). Previous studies have demonstrated that the number of donor T_RM_ cells decreases over time after transplantation, while recipient peripheral immune cells infiltrate the transplanted organ and acquire a tissue-resident phenotype (7, 9). In addition to T_RM_ cells, the tissue-resident immune cell compartment of healthy human kidneys also contains innate and innate-like TRL populations that contribute to local immune surveillance (1, 2). However, evidence of the existence of innate and innate-like TRL populations in kidney transplants is limited (10, 11).

Few studies have investigated the functionality of TRLs in transplanted organs. It has been hypothesized that recipient T_RM_ cells have a deleterious function in the allograft, as reflected by the observation that recipient T_RM_ cells can induce allograft rejection in mice kept in sterile conditions (12, 13). In contrast, donor T_RM_ cells are thought to protect against alloimmune responses, as reflected in the association between the persistence of donor T_RM_ cells in lung and intestinal transplants and reduced rejection events (14, 15). Currently, it is unclear if donor or recipient T_RM_ cells in the immunosuppressed kidney transplant provide local protection against pathogens.

This study presents a comprehensive analysis of donor and recipient TRLs derived from kidney transplant nephrectomies to provide a landscape of tissue-resident immune cells in kidney transplants and explore their potential significance in antiviral immunity.

## Results

### Innate and adaptive TRL populations of donor and recipient origin reside within kidney transplants.

**Figure 1:**
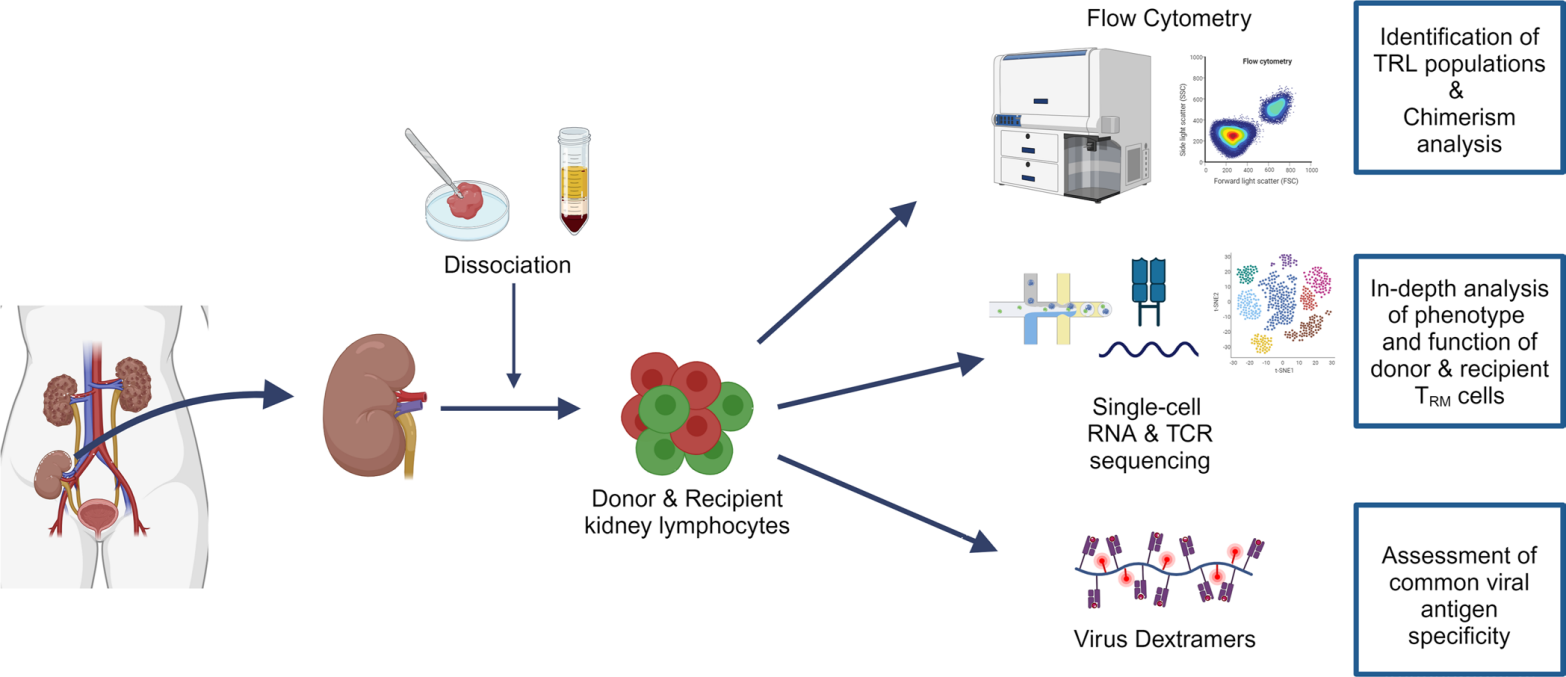
Schematic outline of the study. Schematic showing all experiments conducted in this study. Kidney transplant nephrectomies were collected, and donor and recipient lymphocytes were obtained by mincing the tissue, performing enzymatic digestion, and following a standard Ficoll procedure. The characteristics and functional state of donor and recipient tissue-resident lymphocytes were studied using flow cytometry, virus dextramer staining, and paired scRNA- and TCR-Seq.

In this study, the characteristics and antiviral specificity of TRLs in 24 kidney transplant nephrectomy specimens were studied, and the differences between TRLs of donor and recipient origin were investigated (Figure 1, Supplemental Figure 1, and Supplemental Table 1; supplemental material available online with this article; https://doi.org/10.1172/jci.insight.172681DS1). Most of the kidney transplant nephrectomies were performed because of untreatable rejection (67%), with a median time between transplantation and explantation of 779 days (Supplemental Figure 1 and Supplemental Tables 1 and 2).

**Figure 2:**
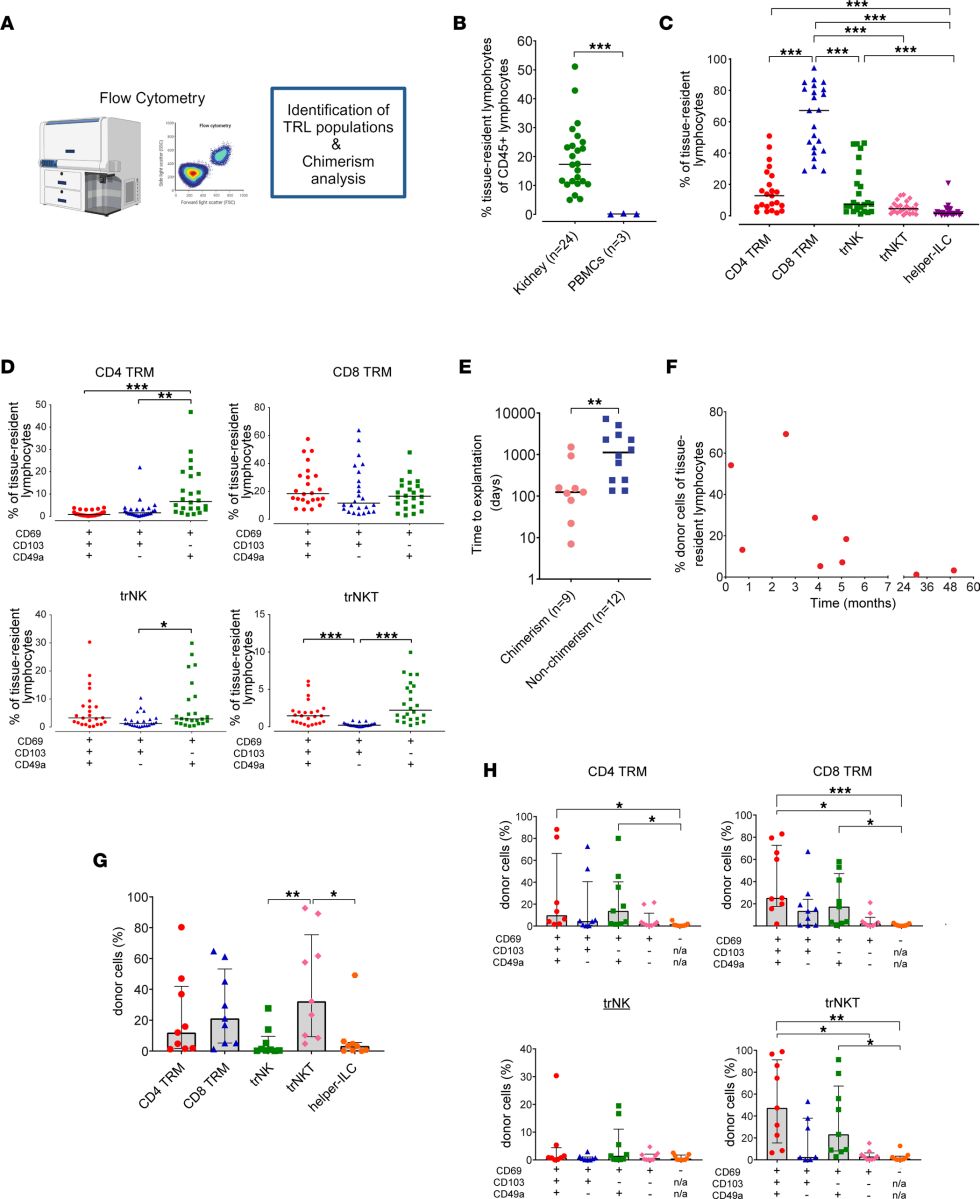
Donor and recipient chimerism of innate(-like) and adaptive TRLs in kidney transplants. (**A**) Flow cytometric analysis to identify donor and recipient TRL populations. (**B**) Frequency of TRLs among CD45^+^ lymphocytes in kidney transplant nephrectomies (*n* = 24) and in PBMCs (*n* = 3). (**C** and **D**) Frequency of TRL subpopulations among total TRLs (**C**) and the differential expression of tissue-resident markers CD69, CD103, and CD49a (**D**) in kidney transplant nephrectomies (*n* = 24). (**E**–**H**) Chimerism analysis of TRLs in kidney transplant nephrectomies (*n* = 21). Comparison of time between transplantation and explantation between chimeric (*n* = 9) and nonchimeric (*n* = 12) samples (**E**). Spearman’s correlation (ρ) between the proportion of donor cells among TRLs in chimeric kidneys (*n* = 9) and the time between transplantation and explantation (ρ = −0.75; *P* = 0.025) (**F**). Frequency of donor cells among TRL subpopulations (**G**). Frequency of donor cells in TRL subpopulations defined by the expression of canonical tissue-resident markers (**H**). Bars represent the median (**B**–**E**). Bar plots represent the median and IQRs (**G** and **H**). CD103 and CD49a expression was not examined in CD69^–^ populations (n/a; **H**). The Mann–Whitney U test was used to compare 2 groups (**B** and **E**). The Kruskal–Wallis test with Dunn’s multiple comparison was used to compare more than 2 groups (**C**, **D**, **G**, and **H**). *P < 0.05, **P <0.01, and ***P < 0.001.

We aimed to investigate, using multiparameter flow cytometry, which TRL populations reside in kidney transplants (Figure 2A, Supplemental Figure 2, and Supplemental Tables 3 and 4). The proportion of lymphocytes with a tissue-resident phenotype in explanted kidney transplants was 17.3% (range: 5.0%–51.1%), whereas 0.07% of peripheral blood lymphocytes had this phenotype (range: 0.04%–0.33%; Figure 2B). CD4^+^ and CD8^+^ T_RM_ cells were detected, and the majority expressed CD69 and CD49a with or without CD103 (Figure 2, C and D). Tissue-resident NK (trNK) and NKT (trNKT) cells were also identified in kidney transplants; however, these cells were less abundant than T_RM_ cells, and CD49a expression was also predominant in these populations (Figure 2, C and D). Helper innate lymphoid cells (ILCs) were also detected but represented a very small proportion of the total TRL compartment (Figure 2C).

The origin of TRLs was determined using human Abs specifically targeting recipient or donor HLA class I (Supplemental Table 4). For 21 of the 24 included kidneys, Abs specific to the mismatch between the donor and recipient were available. TRLs of donor origin were present in 9 of 21 (43%) kidney explants (Figure 2E), whereas TRLs of recipient origin were identified in all kidney specimens. The time between transplantation and explantation was significantly longer in nonchimeric than in chimeric samples, with a median of 1125 (IQR: 242–2798) and 124 (IQR: 51–543; *P* = 0.005; Figure 2E) days, respectively. An inverse correlation was observed among the chimeric samples between the time to explantation and the proportion of donor cells (Spearman’s ρ = −0.75; *P* = 0.025; Figure 2F).

After having demonstrated local chimerism, the proportion of donor cells within each TRL subpopulation was investigated. The lowest proportion of donor cells was observed in the innate trNK and helper ILC populations (0.9% and 3.3%, respectively; Figure 2G). Donor cells were predominantly among trNKT and CD8 T_RM_ cells (32.3% and 21.2%, respectively; Figure 2G), most of which expressed CD69, CD103, and CD49a (Figure 2H). A small number of donor cells was detected in the CD69^+^CD103-CD49a^–^ TRL, CD69^–^ trNK, and CD69^–^ trNKT cell populations; however, none were detected in the CD69^–^ CD4 T_RM_ and CD69^–^ CD8 T_RM_ populations (Figure 2H). This finding indicates that the majority of TRLs express CD69, CD103, and/or CD49a, and that a minority of resident lymphocytes may also express other markers responsible for their resident state.

Thus, these findings demonstrate that innate(-like) and adaptive TRL populations exist within kidney transplants, and that these TRLs generally express a combination of CD69, CD103, and CD49a. Additionally, chimerism occurs in the innate(-like) and adaptive TRL populations, and most donor cells coexpress CD69, CD103, and CD49a.

### Distinct phenotypical and functional T_RM_ subpopulations are present in kidney transplants.

**Figure 3:**
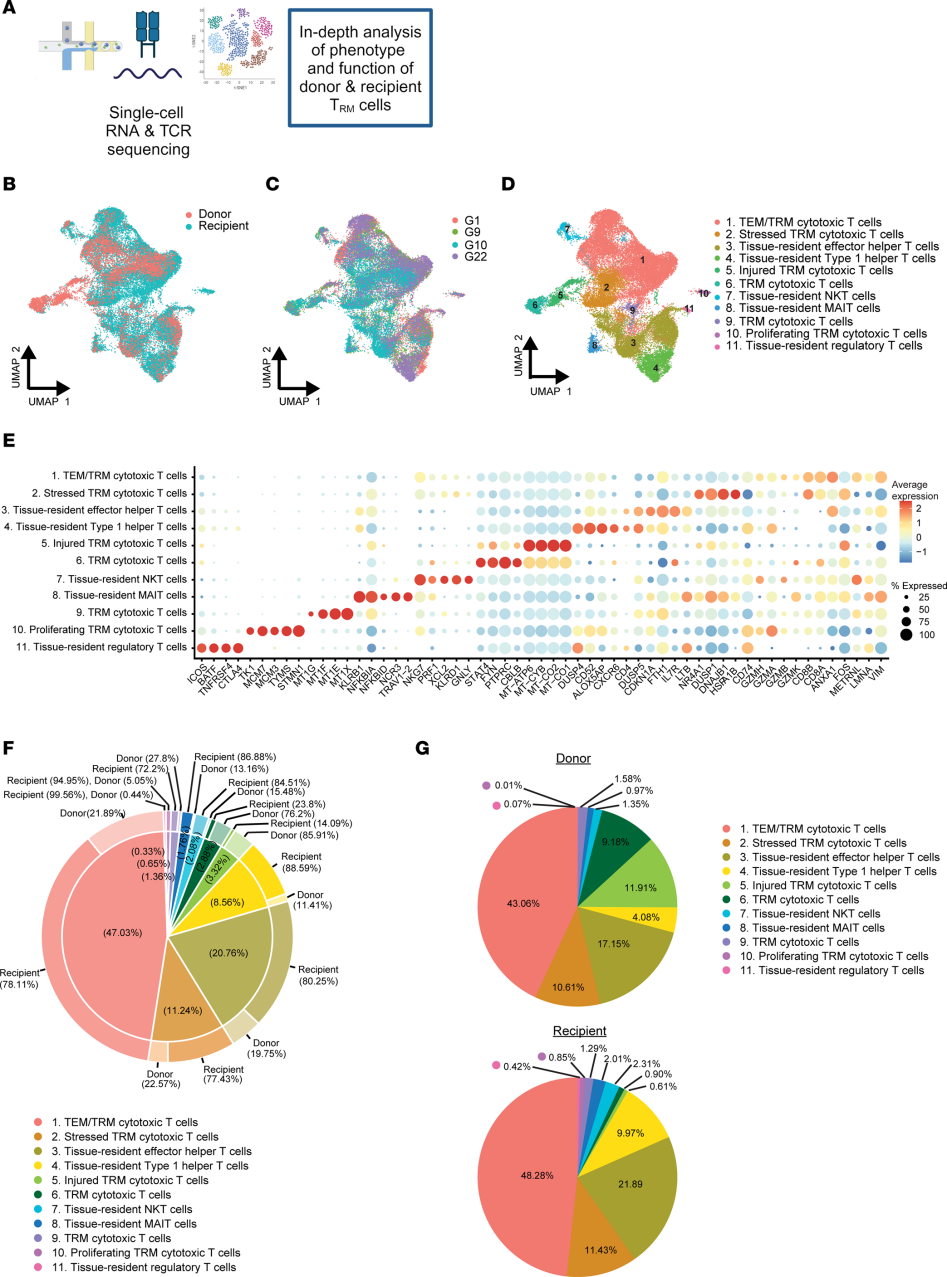
Heterogeneous subpopulations of donor and recipient T_RM_ cells discovered through scRNA-Seq. (**A**) Paired scRNA- and TCR-Seq of chimeric kidney transplant nephrectomies (*n* = 4). (**B**) UMAP showing the distribution of donor and recipient cell origin. (**C**) UMAP showing the distribution of samples (*n* = 4; study sample identifiers G1, G9, G10, and G22) after the anchor’s integration. (**D**) UMAP showing the 11 different cell clusters. Annotation of clusters included the use of CellTypist (57). (**E**) Dot plot showing the most significant DEGs among the cell clusters. (**F**) Pie chart showing the relative size of the cell clusters and the proportion of donor- and recipient-derived cells within each cluster. (**G**) Pie charts showing the proportion of each cell cluster among donor and recipient T_RM_ cells.

An in-depth analysis of the phenotype and functional characteristics of donor and recipient T_RM_ cells was performed through combined single-cell transcriptomic analysis and T cell receptor (TCR) sequencing with sorted CD4^+^ and CD8^+^ T_RM_ cells from 4 chimeric samples (Figure 3A Supplemental Figure 1, and Supplemental Tables 4 and S). A total of 35,309 cells were included, of which 8443 (24%) were of donor origin and 26,866 (76%) were of recipient origin (Figure 3, B and C, and Supplemental Figure 3).

Eleven cell clusters were identified (Figure 3D). Tissue residency of the cell clusters was confirmed by CellTypist and by examination of T_RM_ markers in the scRNA Seq data (Supplemental Figure 4) Six of the 11 clusters were CD8^+^ T_RM_ cytotoxic cell clusters (67% of the total population) and included activated, stressed, injured, and proliferating CD8^+^ T_RM_ cytotoxic cells (Figure 3E). For 2 CD8^+^ TRM cytotoxic cell clusters (clusters 6 and 9), the precise functional and phenotypic implications of the identified differently expressed genes (DEGs) could not be delineated; therefore, we referred to these clusters with a more general designation. Three clusters contained CD4^+^ T_RM_ cells (30% of the total population) and included tissue-resident effector Th cells, tissue-resident Th1 cells, and tissue-resident T_regs_ (Figure 3E). Two innate-like T cell populations were also detected (4% of the total population) and included trNKT cells with an activated and cytotoxic phenotype and activated tissue-resident mucosal-associated invariant T (MAIT) cells (Figure 3E).

Cells of recipient origin were dominant in most clusters, due to the higher number of recipient cells overall (Figure 3F); however, the CD8^+^ T_RM_ cytotoxic cells in clusters 5 and 6 were predominantly donor derived (86% and 76% donor cells, respectively; Figure 3F). These donor-enriched clusters included CD8^+^ T_RM_ cytotoxic cells expressing genes related to injury (cluster 5) and involved in cell activation (cluster 6; Figure 3E). We also investigated the proportion of each cell cluster among the total donor and total recipient compartments. Remarkably, the proportions of most cell clusters were comparable between donor and recipient samples (Figure 3G).

To gain increased insight into the phenotype of donor versus recipient T_RM_ cells, we applied DEG and pathway analyses. Stress-induced genes were significantly upregulated in donor T_RM_ cells versus recipient T_RM_ cells, whereas genes associated with cellular activation (e.g., *CD52* and *LTB*) were upregulated in recipient T_RM_ cells (Supplemental Figure 5). The functional pathway analysis showed that the ribosome pathway was most significantly upregulated in recipient T_RM_ cells, whereas the IL-17 pathway was most significantly upregulated in donor T_RM_ cells (Supplemental Figure 5 and Supplemental Table 6).

Thus, donor- and recipient-derived CD4^+^ and CD8^+^ T_RM_ cells show no similarities in their transcriptomic profiles, including activated, injured, proliferating, and effector signatures. However, donor T_RM_ cells appear to encounter more cellular stress, whereas recipient T_RM_ cells appear to be in a more activated state.

### Evidence for shared TCR specificities between donor and recipient T_RM_ cells.

Single-cell TCR αβ sequencing data were incorporated into the single-cell transcriptome data to understand the TCR composition of donor and recipient T**_RM_** cells. TCR sequences were recovered from 28,879 cells (82% of the total population). Sixty-two percent of cells expressed at least 1 α chain paired with 1 β chain (Supplemental Table 7).

**Figure 4:**
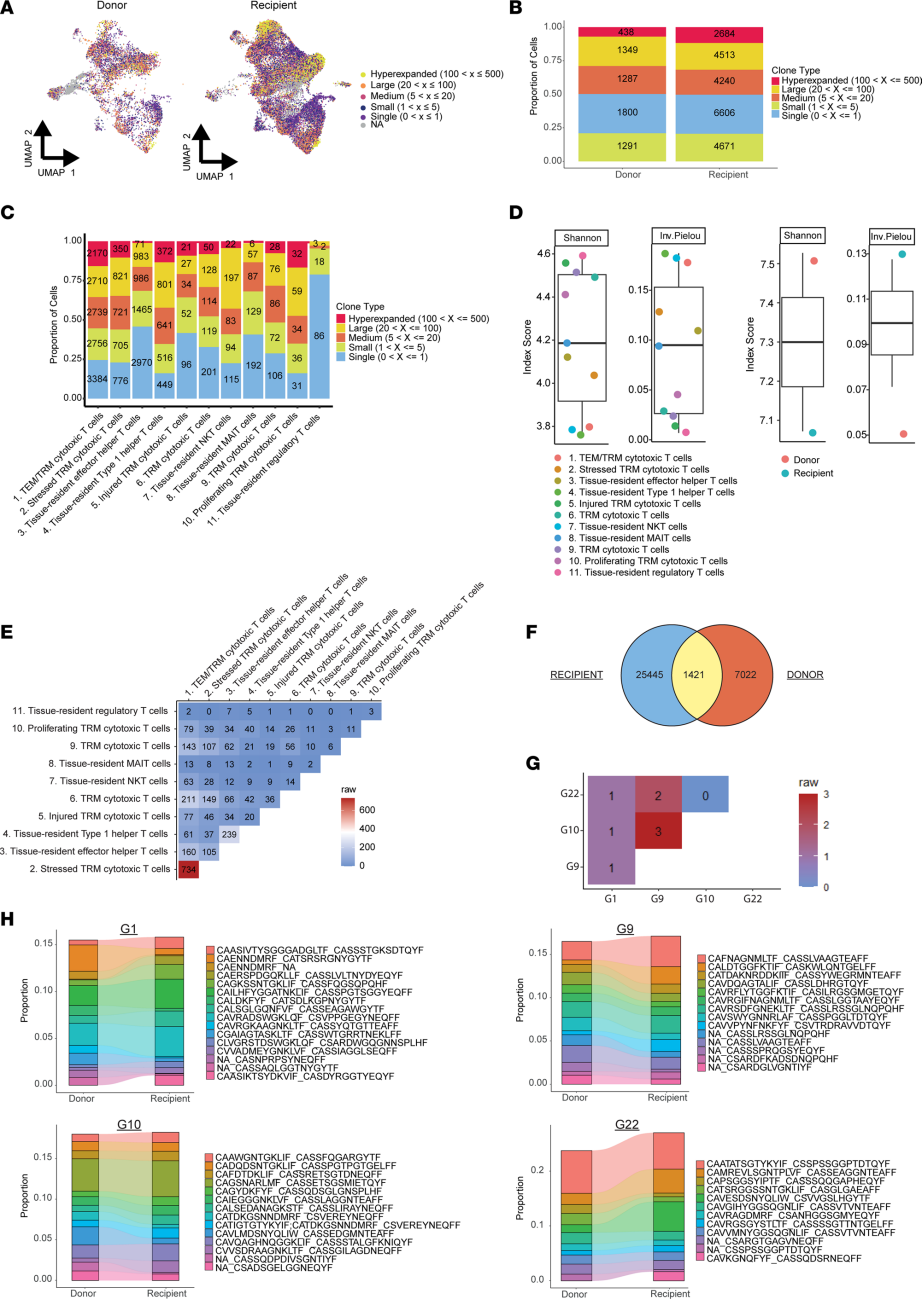
Analysis of TCR clonality of donor and recipient T_RM_ cells. Single-cell TCR-Seq was performed in parallel with the scRNA-Seq of chimeric kidney transplant nephrectomy samples (*n* = 4; study sample identifiers G1, G9, G10, and G22). (**A**) UMAP visualization of TCR expansion in donor (left) and recipient (right) T_RM_ cells. Different colors represent different degrees of TCR expansion. The range between the parentheses indicates the frequency of the expressed clonotype across the samples (×). (**B** and **C**) Clonotype size distribution across donor and recipient T_RM_ cells (**B**) and per cell cluster (**C**). The proportion of clonotype size is indicated on the *y* axis. The number of cells within each clonotype size group is shown in the graph. (**D**) Shannon and inversed Pielou’s scores are shown for different cell clusters (left) and for donor and recipient T_RM_ cells (right). Box-and-whisker plots show the median, IQR, minimum, and maximum values (excluding outliers). (**E** and **G**) Analysis of clonal overlap between different cell clusters (**E**) and samples (**G**). Numbers indicate the absolute number of shared unique clonotypes. (**F**) Venn diagram showing the unique clonotypes for recipient and donor T_RM_ cells. The overlap indicates the number of shared unique clonotypes. (**H**) The top 10 unique clonotypes among donor and recipient T_RM_ cells for each sample. The proportion of clonotypes is indicated on the *y* axis. Each color represents a unique clonotype. Colored bands connect the shared bar plot segments between donor and recipient. The sequence reads of the clonotypes are shown next to the graphs.

Clonal expansion and loss of TCR diversity have been linked to alloreactive T cells (16). Therefore, we postulated that recipient T_RM_ cells would have higher TCR clonality than donor T_RM_ cells. Indeed, hyperexpanded clonotypes (defined as 100–500 cells with an identical TCR sequence) were mostly of recipient origin (86%; Figure 4, A and B). The proportion of hyper-expanded clonotypes was twice as high in recipient versus donor cells (10% *vs*. 5%, respectively; Figure 4B and Supplemental Table 8). The proportion of hyperexpanded clonotypes within each cell cluster was highest for effector memory T cells (T_EM_)/T_RM_ (cluster 1, 16%) and proliferating T_RM_ (cluster 10, 17%) cytotoxic cells and lowest for the tissue-resident T_regs_ (cluster 11, 0%; Figure 4C and Supplemental Table 9). The Shannon entropy and inverse Pielou’s scores were calculated for each cell cluster as measures of clonal diversity and evenness, respectively (Figure 4D). T_EM_/T_RM_ cytotoxic cells (cluster 1), tissue-resident Th1 cells (cluster 4), and trNKT cells (cluster 7) had the lowest TCR diversity and had an uneven distribution (represented by low Shannon entropy and high inverse Pielou’s scores; Figure 4D). Recipient T_RM_ cells were also compared with donor T_RM_ cells. Recipient T_RM_ cells had a lower TCR diversity and were more unevenly distributed (Figure 4D).

Next, the clonal overlap between different cell clusters and between donor and recipient T_RM_ cells was analyzed. Stressed T_RM_ cytotoxic cells (cluster 2) and T_EM_/T_RM_ cytotoxic cells (cluster 1; Figure 4E) shared the most clonotypes. Tissue-resident Th1 and effector Th cells also shared a substantial number of clonotypes (Figure 4E). Donor T_RM_ cells contained 3,340 unique clonotypes (54% of the total donor T_RM_ cell population with available TCR data), and recipient T_RM_ cells exhibited 9447 unique clonotypes (42% of the total recipient T_RM_ cell population with available TCR data). Among these unique clonotypes, 1421 were shared between donor and recipient T_RM_ cells (43% of donor and 15% of recipient unique clonotypes; Figure 4F). However, almost no clonotypes were shared between samples (Figure 4G), which aligns with the low proportion of clonotype sharing among memory T cells observed in individuals at random (17). The high proportion of shared clonotypes between donor and recipient T_RM_ cells might result from the fact that kidney donors and recipients are partially HLA matched. HLA mismatches between recipients and corresponding donors are provided in Supplemental Table 10.

Finally, the 10 most prevalent donor and recipient clonotypes were examined. Because of the low proportion of clonotype sharing among the 4 samples (Figure 4G), these clonotypes were examined in each sample separately. We hypothesized that the recipient T_RM_ compartment would contain an alloreactive cell population comprising hyperexpanded clones among the 10 most prevalent recipient clonotypes not shared with donor T_RM_ cells. However, almost all prevalent donor and recipient clonotypes were shared within each sample, suggesting that these clonotypes do not express alloreactive TCRs (Figure 4H).

Overall, these findings demonstrate that recipient T_RM_ cells have higher clonality than do donor T_RM_ cells and that recipient and donor T_RM_ cells share numerous clonotypes.

### Both donor and recipient T_RM_ cells have predicted TCR specificities against viral pathogens.

A string-search analysis was performed to reveal the antigen specificity of donor and recipient T_RM_ cells. First, 4 databases containing CDR3 α and β sequences with known TCR antigen specificities were searched for a match within the 10 most prevalent donor and recipient clonotypes per sample (Figure 4H and Supplemental Table 11) (18–21). The antigen specificity was predicted for 13 TCR sequences and included specificities against CMV, EBV, influenza, HIV, SARS-related coronavirus 2 (SARS-CoV2), *Mycobaterium* *tuberculosis*, and yellow fever virus (YFV; Supplemental Table 11). Multiple matches were found for some TCR sequences.

**Figure 5:**
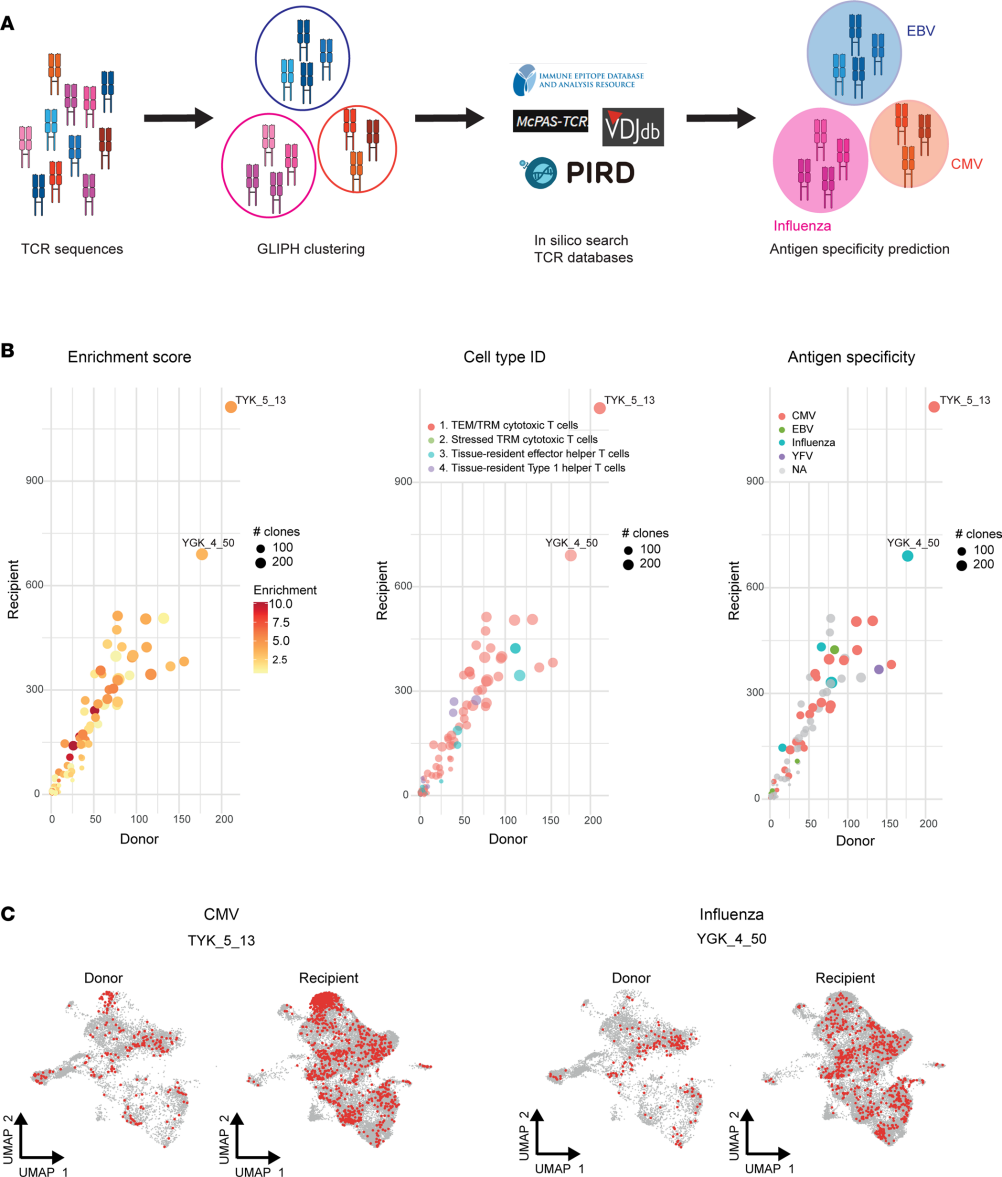
Prediction of antigen specificity of donor and recipient T_RM_ cells. (**A**) Schematic of GLIPH2 clustering of TCR sequences and string-search analysis for antigen specificity prediction. (**B**) GLIPH2 clusters according to enrichment score (left), predominant cell cluster (middle), and predicted antigen specificity (right). Each dot represents a GLIPH2 cluster. The number of recipient and donor T_RM_ cells within each cluster is plotted on the *y* and *x* axes, respectively. (**C**) UMAP visualization of the top 2 GLIPH2 clusters showing the distribution of corresponding donor and recipient cells across different cell clusters.

Next, grouping of lymphocyte interactions by paratope hotspots (GLIPH2) was used to perform a more comprehensive analysis of TCR specificities (Figure 5A) (22). GLIPH2 clusters TCR sequences predicted to have shared antigen specificity. A total of 139 distinct GLIPH2 clusters were discovered (Supplemental Table 12). Surprisingly, no specific clusters for donor or recipient cells were found; however, all clusters were shared between donor and recipient samples (Figure 5B). The dominant cells in most clusters were T_EM_/T_RM_ cytotoxic cells (Figure 5B and Supplemental Table 12). A string-search analysis was performed using 4 TCR databases to predict the antigen specificity of the GLIPH2 clusters. The antigen specificity was only assigned to clusters for which at least 1 match was found for more than 20% of unique clonotypes or for more than 25 unique clonotypes to increase the certainty of prediction analysis. The antigen specificity could be assigned using this cutoff in 40 clusters (Figure 5B). Most clusters were predicted to target CMV. Other predicted specificities were against EBV, influenza, and YFV (Figure 5B and Supplemental Table 12). The top 2 clusters were predicted to have specificity against CMV (TYK_5_13) and influenza (YGK_4_50) and contained mostly T_EM_/T_RM_ cytotoxic cells of donor and recipient origin (Figure 5, B and C). Together, these findings suggest that donor and recipient T_RM_ cells share antigen specificities predicted to react against viral pathogens.

### EBV, CMV, BK polyomavirus, and influenza A–specific T_RM_ cells of donor and recipient origin reside in transplanted kidneys.

**Figure 6:**
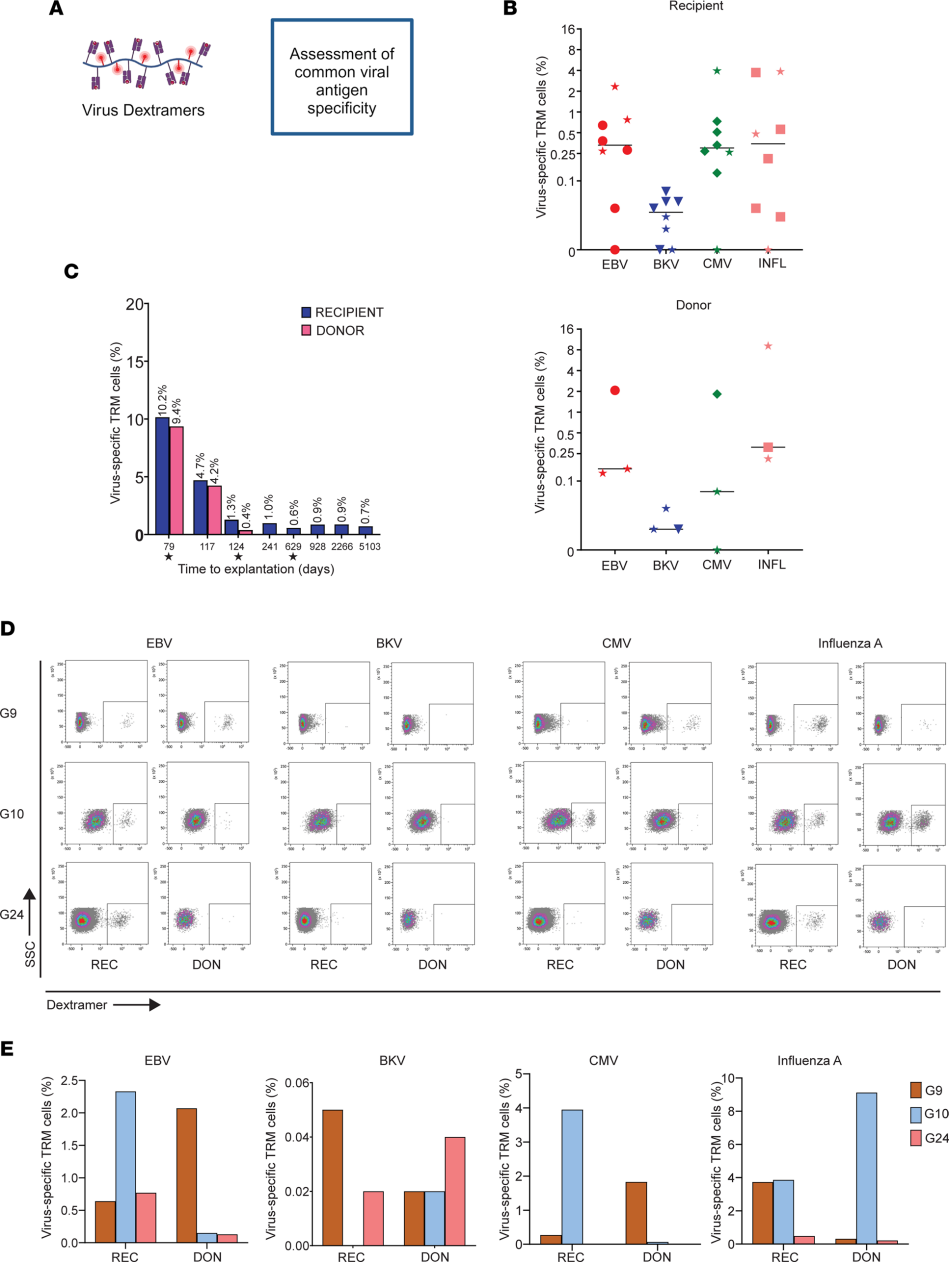
Virus specificity of donor and recipient CD8 T_RM_ cells. (**A**) Virus dextramer staining and flow cytometric analysis to examine the virus specificity of CD8^+^ T_RM_ cells using EBV, BKV, CMV, and influenza A dextramers in HLA-A2^+^ kidney transplant nephrectomies (*n* = 8). (**B**) Positive dextramer staining of recipient and donor T_RM_ cells against EBV, BKV, CMV, and influenza A (INFL). Bars represent the median. A star indicates patients who were treated with alemtuzumab prior to explantation. (**C**) Total proportion of virus-specific cells among donor and recipient T_RM_ cells in each sample. The time between transplantation and explantation is annotated on the *x* axis. A star indicates patients who have been treated with alemtuzumab prior to explantation. (**D**) Flow cytometry plots showing dextramer staining of donor and recipient T_RM_ cells among the chimeric samples (study sample identifiers G9, G10, and G24). (**E**) Graphs showing the proportion of EBV-, BKV-, CMV-, and influenza A–specific donor (DON) and recipient (REC) T_RM_ cells among the chimeric samples (G9, G10, and G24).

Virus dextramer experiments were performed to confirm the observation that donor and recipient T_RM_ cells have TCR specificities against common viral pathogens. To this end, kidney lymphocytes were stained with EBV, CMV, BK polyomavirus (BKV), and influenza A peptide-loaded HLA-A*02:01 dextramers (Figure 6A, Supplemental Figure 6, and Supplemental Tables 4 and 13). Among the 21 explants samples for which donor and recipient HLA-specific Abs were available, 8 were from HLA-A*02^+^ recipients and donors and could be used for these experiments (Supplemental Figure 1 and Supplemental Table 10). The EBV and CMV serostatuses of the donors and recipients at the time of explantation are provided in Supplemental Table 14.

The kidney CD8^+^ T_RM_ cells of recipient origin showed virus specificity against all 4 viral antigens in the same range as the donor CD8^+^ T_RM_ cells (Figure 6B). In the 3 chimeric samples, we observed that the total proportion of virus-specific T_RM_ cells was comparable between donor and recipient; however, there were significant changes in the proportions of T_RM_ cells specific for each virus (Figure 6, C–E). The highest proportion of virus-specific recipient T_RM_ cells was observed in the 2 samples from patients with the shortest time to explantation (97 and 117 days; Figure 6C). Infections related to these viruses after transplantation were documented for only 2 patients (Supplemental Table 14). As a result, we could not perform a correlation analysis between posttransplant infections and the proportions of virus-specific T_RM_ cells. Interestingly, 1 of these patients, G24, experienced a primary CMV infection around the time of explantation in the absence of CMV-specific T_RM_ cells (Figure 6, D and E, and Supplemental Table 14).

Last, a potential effect of T cell depletion from alemtuzumab treatment on the virus-specific TCR repertoire was investigated. No difference was observed in proportions of virus-specific T_RM_ cells of patients with a history of alemtuzumab treatment compared with those without (Figure 6, B and C, and Supplemental Figure 1).

These findings suggest that CD8 T_RM_ cells of both donor and recipient origin can potentially mount antiviral responses in transplanted kidneys, which aligns with the findings obtained from the GLIPH2 clustering and string-search analyses.

## Discussion

TRLs are increasingly recognized for their crucial role in pathogen defenses under physiologic circumstances. However, their characteristics and functions in the transplanted organs of immunosuppressed recipients are not fully understood (3, 5, 23, 24). In the present study, we demonstrated that kidney transplants contain a diverse population of both donor and recipient TRLs. Among those are donor and recipient T_RM_ cells that have a substantial resemblance in transcriptomic profiles and a highly similar TCR repertoire, demonstrating specificity for common viral pathogens.

TRLs are crucial for local antimicrobial immunity in mouse and human studies focusing on T_RM_ cells in the lung, liver, intestine, brain, and skin (24–28). However, studies investigating the role of TRLs in protective immunity in human kidneys are limited. One study demonstrated that peritumor and renal carcinoma tissue contains EBV-, CMV-, BKV-, and influenza-specific T_RM_ cells (29). Another previous investigation revealed BKV-specific T_RM_ cells in kidney transplant tissue, with increased frequencies in kidneys with BKV-associated nephropathy (BKVAN) versus kidneys without BKVAN (30). The origin (i.e., donor or recipient) of these BKV-specific T_RM_ cells was not examined. The present investigation demonstrates that kidney transplants contain a diverse population of T_RM_ cells of both donor and recipient origins and that these cells have virus specificity. The proportions of virus-specific T_RM_ cells detected with virus dextramer staining are comparable to those of T_RM_ cells against these same viral antigens in peritumor kidney tissue in previous studies (29). We only detected small numbers of BKV-specific T_RM_ cells in the kidney transplants, comparable with the proportions of BKV-specific T cells observed in kidney transplants without BKVAN (30). Thus, our findings indicate that the presence of virus-specific T_RM_ cells in the donor organ is, at least partly, maintained under conditions of chronic immunosuppression.

The absence of virus-specific T_RM_ cells in the allograft of patient G24 during a primary CMV infection is an intriguing observation. It is plausible that the allograft harbored the recipient’s CMV-specific T cells that had not yet differentiated into T_RM_ cells at that time. This interpretation aligns with earlier studies suggesting that the formation of T_RM_ cells generally follows after the clearance of infection (31). Alternatively, it is conceivable that the allograft of patient G24 did contain virus-specific T_RM_ cells that our detection method might have missed because the dextramer used is specific to just 1 CMV epitope within the context of HLA A2*01 molecules.

The finding that influenza A–specific T_RM_ cells also reside within transplanted kidneys contradicts the idea that T_RM_ cells are generated in response to local antigen stimulation. The local presence of influenza A viral antigens within kidneys has been reported only in a few cases of the pandemic H1N1 influenza variant and never for seasonal variants (32–34). Therefore, the local presence of influenza A viral antigens within the kidneys studied in this investigation and in a previous study does not seem a plausible explanation for the influenza A–specific T_RM_ cells residing in the tissue (29). Alternatively, it has been reported that antigen-independent inflammation can trigger the infiltration and generation of virus-specific T_RM_ cells, which may explain the presence of these influenza A–specific T_RM_ cells within the kidney (35). Considering that T_RM_ cells can also actively re-enter the circulation and differentiate into T_EM_ cells, one can speculate that the reservoir of influenza A–specific T_RM_ cells in the kidney may assist in maintaining distant antiviral immunity through this process of retrograde migration (24, 36).

Last, cross-reactivity of influenza A–specific T_RM_ cells against other locally present antigens within the kidney may also occur (37, 38). Whether influenza A–specific T_RM_ cells exist in other solid organs except the lungs is unknown (25). Our data also suggest that SARS-CoV2–specific T_RM_ cells reside within kidney transplants. In contrast to influenza A, SARS-CoV2 viral antigens have commonly been observed within kidney tissue (34). Remarkably, the 3 TCR specificities predicted to target SARS-CoV2 were observed in 2 prepandemic samples (study sample identifiers G9 and G10). Prepandemic SARS-CoV2–specific T_RM_ cells had also been observed in healthy donor lungs and might result from cross-reactivity between SARS-CoV2 and seasonal coronaviruses (39).

The currently accepted concept is that recipient T_RM_ cells play a major role in solid-organ allograft rejection, as evidenced in both mouse and human studies (12–15, 40). Likewise, the elimination of recipient T_RM_ cells has been suggested as a future therapeutic goal to reduce the risk of allograft rejection. Although we recognize the likely presence of recipient alloreactive T_RM_ cells within the kidney explants, we did not investigate alloreactivity of T_RM_ cells in this study. In contrast, our study provides evidence that recipient T_RM_ cells may not be solely harmful, as reflected in the finding that recipient T_RM_ cells harbor a broad TCR repertoire against common viral pathogens. This observation, therefore, provides evidence against the suggestions made for the therapeutic elimination of all T_RM_ cells in transplanted organs, because such an approach will also abrogate the potential favorable antiviral T_RM_ immune responses.

Our study differs from previous human studies in that it does not include acute rejection biopsy samples but rather end-stage kidney transplant tissue with or without rejection. T_RM_ cell plasticity may cause variations in the T_RM_ cell population (i.e., alloreactive or virus-specific T_RM_) during acute allograft rejection versus that in end-stage donor organs (24, 41, 42). Additionally, recipient T_RM_ cells could be both beneficial and harmful, due to the occurrence of heterologous immunity between alloreactive and virus-specific T cells (37, 43). Future research is essential to further elucidate the extent to which T_RM_ cells exhibit alloreactivity and how this relates to their antiviral properties.

We showed through transcriptomic analysis that donor and recipient T_RM_ cells have clonal similarities and exhibit other shared features. The donor and recipient T_RM_ compartments contained largely similar proportions of diverse subpopulations. Most T_RM_ cells in both compartments were CD8^+^ T_RM_ cells displaying an effector phenotype, which aligns with findings in other organ transplant studies (7, 14). In contrast, 2 CD8 T_RM_ subpopulations were almost exclusively donor derived and contained large numbers of injured cells (as reflected in the increased number of mitochondrial genes), which may represent the gradual loss of donor T_RM_ cells. This observation suggests that the gradual loss of donor T_RM_ cells in kidney transplants is, at least partly, a consequence of injury that results in cell death. Alloreactive immune responses orchestrated by infiltrating recipient immune cells are likely responsible for this injury (44). Alternatively, the gradual loss of donor T_RM_ cells may be a result of the limited lifespan of T_RM_ cells and the process of retrograde migration (24, 36). Overall, time is a major factor in the gradual loss of donor cells, as reflected in the strong time-dependent donor cell count observed using flow cytometry, which aligns with previous observations (7).

Local immune surveillance is not only executed by T_RM_ cells but also relies heavily on the interplay of both innate(-like) and adaptive TRLs (2, 4, 5). We observed that in the unique immunological environment of a kidney transplant (i.e., foreign immune system and immunosuppressive drugs), both innate(-like) and adaptive TRLs reside within kidney transplants. We identified previously characterized subsets of TRLs in kidney transplants (CD4^+^ T_RM_, CD8^+^ T_RM_, and MAIT cells) and subsets not previously found in kidney transplants (trNK, trNKT, and helper ILCs) (7, 8, 10). The expression of CD49a and not CD103 was previously recognized to be predominant in kidney T_RM_ cell populations but has not been examined in earlier kidney transplant studies (45). We show that most T_RM_ cells and other TRLs in kidney transplants express CD69 and CD49a with or without CD103, confirming the importance of CD49a as a resident marker in TRLs. Chimerism was observed in all TRL populations but to a different degree across populations. Fewer donor cells were found in innate than in adaptive TRL populations, which aligns with previous observations in intestinal transplants (15, 46). This finding provides evidence against the hypothesis that innate TRLs reside longer within the allograft after transplantation than T_RM_ cells, because the formation and maintenance of innate TRLs are independent of antigen-specific stimuli, and they have local self-renewal capacities (2, 6, 47, 48). This finding may be explained by the observation that T_RM_ cells also have self-renewal properties, supported by the identified cluster of proliferating T_RM_ cells in our single-cell analysis. Additionally, previous research has shown that donor T_RM_ cells can proliferate locally in a mouse liver transplant model (49). Moreover, the formation and maintenance of T_RM_ cells are not, per se*,* antigen dependent, suggesting that donor T_RM_ cells may persist in tissues irrespective of antigen-specific stimuli (35).

This study has several limitations. First, our results are derived from explanted, severely damaged, nonfunctioning kidney transplants. Therefore, we cannot extrapolate our findings to stable kidney transplants or those that encounter early acute rejection. Second, dissociation stress is an unavoidable consequence of the methods used to harvest lymphocytes from tissue. Dissociation stress can lead to cell-specific upregulation of stress-induced genes in single-cell RNA-Seq (scRNA-Seq) (50). However, because all included cells were T_RM_ cells, we believe that the upregulation of stress-induced genes in T_RM_ cytotoxic cells of cluster 5 reflects a biological stress response rather than dissociation stress. Last, a note of caution should be made for the TCR antigen prediction results. Although we tried to limit the uncertainty in the predictions as much as possible, in silico–based antigen prediction should be considered a mere indication of antigen specificity and does not predict TCRs reactivity against allo-HLA molecules. Virus dextramers were used to validate some of the predicted antigen specificities. Additional validation experiments are recommended to substantiate these and other predicted antigen specificities. Moreover, the functional activity of these cells upon antigen exposure should be examined in subsequent studies, potentially through methods like TCR transduction and viral peptide stimulation assays. Last, given the limited size of our dataset and the heterogeneous nature of immunosuppressive regimens, a robust analysis of a potential relationship between immunosuppressive treatment and virus-specific T_RM_ cells was not feasible. This limitation also applies to assessing any potential association between posttransplant infections and the proportion of virus-specific T_RM_ cells.

The current knowledge about TRLs in human solid-organ transplants is limited. Our study provides insights into the composition of TRL subsets in human kidney transplants and identifies that donor and recipient T_RM_ cells both potentially play a role in local viral immune responses. Understanding the role of TRLs in posttransplant immune responses is of high clinical significance because obtaining an optimal balance between preventing alloreactive responses and maintaining protective immunity remains an enormous challenge in daily clinical practice. Therefore, future studies of biopsy specimens obtained over time after transplantation will be essential to fully elucidate the characteristics and functions of donor and recipient TRLs in transplantation and contribute to our understanding of their role in local immune surveillance in the transplanted organ. Future research, involving larger cohorts, could also explore whether patients with a higher proportion of virus-specific T_RM_ cells may have a reduced incidence of viral infections.

## Methods

### Study design.

The overarching goal of this exploratory study was to increase our understanding of the characteristics and virus specificity of donor and recipient TRLs in human kidney transplants. To this end, we used human kidney tissue samples from transplant nephrectomy specimens. The experimental design is summarized in Figure 1.

Kidney tissue was prospectively collected from transplant nephrectomy specimens (*n* = 24) obtained at the Erasmus MC, University Medical Center, between December 2016 and August 2022. The main reasons for removal were untreatable acute or chronic rejection or to provide room for a new donor kidney (Supplemental Table 1 and Supplemental Figure 1). Half of the kidney from each patient was used for routine clinical diagnostic assessments; the other half, after detailed macroscopic analysis, was considered residual material and was included in this study. Transplant nephrectomy specimens were assessed by an experienced nephropathologist (M.C.C.v.G.) using the Banff 2019 classification (44). A summary of these results is provided in Supplemental Table 2. Peripheral blood samples from 3 patients were retrospectively obtained from the Erasmus MC biobank (Supplemental Figure 1). These blood samples were obtained after transplantation and less than 3 months before explantation.

### Isolation of mononuclear cells from kidney tissue and peripheral blood.

Lymphocytes from kidney tissue were obtained through mechanical and enzymatic digestion, as described previously (7). In brief, kidney tissue was dissected into small pieces, followed by incubation with collagenase IV (Serva). A single-cell suspension was obtained using strainers with different pore sizes up to 100 μm. Subsequently, mononuclear cells were harvested through Ficoll density gradient centrifugation (Ficoll-Paque Plus; GE Healthcare). PBMCs were collected from heparinized blood samples using a standard Ficoll procedure (51). Isolated kidney lymphocytes and PBMC samples were cryopreserved at −195°C until further use.

### Flow cytometry analysis.

Isolated mononuclear cells from kidneys and peripheral blood were thawed and stained with the fluorescently labeled Abs listed in Supplemental Table 4 (panel 1) (51). The staining panel included Abs for the characterization of TRLs and HLA-allotype–specific Abs to determine the cell origin (donor or recipient derived). Residency was defined as the coexpression of CD69 plus CD103 and/or CD49a for CD4, CD8, NK, and NKT-like cells (29, 52). No additional resident markers were used for the resident cell type helper ILC. The residency criteria and the gating strategy of cell subsets are provided in Supplemental Table 3 and Supplemental Figure 2, respectively.

Human mAbs against HLA class I discrepancies between donor and recipient were developed at the Leiden University Medical Center (53). These HLA Abs were fluorescently labeled using the Alexa Fluor 488 Conjugation Kit (Fast) – Lightning-Link (Abcam). All other Abs were fluorescently labeled by the manufacturer (Supplemental Table 4). Brilliant Stain Buffer Plus (BD Bioscience) was added to each staining to minimize staining artifacts due to the use of multiple brilliant fluorochromes. Stained samples were measured on a FACSymphony A3 Cell Analyzer (BD Biosciences) and analyzed with Kaluza Analysis 2.1 (Beckman Coulter). Isotype controls were used to set gate limits (Supplemental Table 4).

### Analysis of virus-specific T cells.

The analysis of virus-specific CD8^+^ T_RM_ cells was performed using kidney mononuclear cells from HLA-A*02^+^ patients. The patient HLA-typing information is provided in Supplemental Table 10. A virus dextramer panel (Immudex) was used, which contains dextramers to detect EBV-, CMV-, influenza A-, and BK-virus–specific HLA-A*02:01 T cells and a negative control dextramer (Supplemental Table 13). The EBV and CMV serostatuses of donors and recipients at the time of explantation are provided in Supplemental Table 14. To limit TCR internalization after dextramer staining, cells were first incubated for 30 minutes at 37°C with 50 nM of the protein-kinase inhibitor dasatinib (Sigma-Aldrich) (54, 55). Next, 5 μL of virus dextramer was added, and the cells were incubated for 30 minutes on ice, followed by 20 minutes of staining with the fluorescently labeled Abs listed in Supplemental Table 4 (panel 2). Samples were subsequently measured and analyzed using a FACSymphony A3 Cell Analyzer and Kaluza Analysis 2.1, respectively. Negative controls were used for setting gate limits and background correction (i.e., the negative control signal was subtracted from each positive virus dextramer signal).

### scRNA- and TCR-Seq.

Four chimeric kidney explant samples were used for the single-cell transcriptomic analysis. These 4 samples were selected on the basis of the availability of sufficient cell numbers. Isolated mononuclear cells were thawed and stained with the fluorescently labeled Abs listed in Supplemental Table 4 (panel 3). FACSort of CD4 T_RM_ and CD8 T_RM_ cells was performed using a FACS AriaII cell sorter (BD Biosciences). The sorted cell populations are listed in Supplemental Table 5. A purity check was performed after each FACSort procedure; purity exceeded 90% for all samples.

Libraries were prepared on a chromium controller using the Chromium Next GEM Single Cell 5′ Reagent Kit V2 in combination with the Chromium Single Cell Human TCR Amplification Kit (10x Genomics). In this way, single-cell transcriptomes and immune profiles were generated from the same sample. Next-generation sequencing (26-10-10-90 cycles) of both libraries (gene expression and TCR enriched) was performed on an Illumina NovaSeq 6000 platform. The raw data were processed into FASTQ files. Raw sequences were inspected for quality using FastQC (version 0.11.5; Babraham Bioinformatics). Reads containing sequence information were mapped to the GRCh38 human genome. The generation of BAM files and filtered gene-barcode matrices was accomplished using Cell Ranger Software (version 6.0; 10X Genomics).

### Data analysis.

Data analyses were performed in R (version 4.2.1) (R Foundation for Statistical Computing) with Seurat (version 4.3.0) (56). In the initial preprocessing step, we removed cells expressing fewer than 200 genes, more than 2500 genes, and greater than 5% mitochondrial genes. (Supplemental Figure 3B). Genes expressed in fewer than 3 cells were filtered out. Data were initially normalized for sequencing depth by dividing by the total number of unique molecular identifiers in every cell and then transformed to a log scale for each cell, using the NormalizeData function. Data were then integrated using reciprocal principal component analysis, where anchor genes were the variable genes obtained using the variance-stabilizing transformation method. This step also removed batch effects. Scaling and principal component analysis were performed on the integrated data. Twenty-five principal components were used, covering a 2× SD (95.4%) of the data variance. Cells were then clustered using the shared nearest-neighbor modularity optimization-based clustering algorithm with a resolution of 0.35. Uniform manifold approximation and projection (UMAP) was used for the 2-dimensional representation of the data. Offset populations (i.e. endothelial and distal tubule cells) found within the data were identified using canonical genes and removed. The remaining clusters were reanalyzed following the described pipeline above for further analysis.

Lists containing DEGs within clusters were generated, with a log-fold change greater than 0.25. CellTypist (version 1.3.0) was used for automated cell annotation of the clusters (57). The annotations were then manually confirmed and refined according to the DEGs. DEG and pathway analyses were performed between donor and recipient cells using a Wilcoxon rank-sum test with Bonferroni correction using the Kyoto Encyclopedia of Genes and Genomes 2019 database (58). Dot plots were used to represent gene expression by cluster, where the dot size represents the percentage of cells expressing the gene, and the color scale shows the average nonzero expression on the log2 scale; data were not shown if the percentage of expression was less than 0.01.

Donor and recipient identity deconvolution in scRNA-Seq data was conducted using demuxlet, following the previously published method (59). Briefly, Strelka2 was used to generate variant calling format (VCF) files from the original scRNA BAM files using the human reference genome (GRCh38_CR) and the single nucleotide variant annotator ANNOVAR hg38 (60). Then, VCF files were processed with demuxlet with default values. Identity interpretation was performed using the best files’ output for each sample.

The immune profiling data from the TCR-Seq were analyzed and merged with the scRNA data with scRepertoire (version 1.7.2) (61). The clonal diversity analysis was performed on both chains (TRA and TRB), and 2 metrics were reported (Shannon and inverse Pielou’s scores). The Shannon score is an estimate of clonal diversity; inverse Pielou’s score measures clonal evenness (62, 63). TCR sequences were clustered using local diversity clustering in GLIPH2 (22). GLIPH2 clusters’ antigen-specificity prediction was conducted by matching TCR sequences with VDJdb, PIRD, IEDB, and McPAS-TCR databases (18–21). TCR specificity was assigned only to clusters for which at least 1 match was found for more than 20% of unique clonotypes or for more than 25 unique clonotypes. For single TCR sequences, the antigen specificity was only assigned if the match for either the α or β chains had a Levenshtein distance of 0 or if the match was for the α plus β chain with a maximum Levenshtein distance of 1.

### Statistics.

GraphPad Prism version 9 (GraphPad Software) was used for the statistical analyses. The normal distribution of data was examined with the Kolmogorov–Smirnov test. None of the data were normally distributed; therefore, only nonparametric tests were performed. The Mann–Whitney U test was used to compare 2 groups, and the Kruskal–Wallis test with Dunn’s multiple comparison test was used to compare more than 2 groups. Spearman’s rank correlation (ρ) was used for the correlation between TRL chimerism and the time between transplantation and explantation. *P* values < 0.05 were considered to represent significance. For the chimerism analysis, the proportion of donor cells was calculated only for gated subpopulations with a minimum of 50 cells. Otherwise, the data were excluded from the analysis.

### Study approval.

The study was approved by the Medical Ethical Review Board of the Erasmus MC (approvals MEC-2010-080, MEC-2010-022, and MEC-2020-0791). Residual materials were used in accordance with research not compliant with the Wet medisch-wetenschappelijk onderzoek met mensen (WMO; Medical Research Involving Human Subjects Act) that is regulated by the Dutch Code of Conduct (Federa). All experiments were performed in accordance with relevant guidelines and regulations as described by our institution. All patients gave written informed consent. No organs were procured from (executed) prisoners.

### Data availability.

All single-cell mRNA- and TCR-Seq data generated in this study have been deposited in the NCBI’s Gene Expression Omnibus database database (GEO GSE242909). All other data associated with the manuscript and supplemental material are provided in the Supporting Data Values.

## Author contributions

DMHP, DAH, MJH, and CCB designed the research; DMHP, MD, MCCVG, and RCM were involved in sample inclusion. SH, TPPVDB, GMGMV, HTM, and EMJB provided specific expertise on methodology and data interpretation. SH provided reagents. DMHP, MD, and EMJB performed the experiments. DMHP and HTM performed the data analyses. DAH, RCM, and MCCVG provided clinical expertise and assistance with clinical data analyses. DMHP wrote the original draft. DMHP, HTM, DAH, EMJB, MCCVG, SH, RCM, GMGMV, MJH, and CCB reviewed and edited the manuscript.

## Supplementary Material

Supplemental data

Supplemental table 10

Supplemental table 11

Supplemental table 12

Supplemental table 14

Supplemental table 6

Supporting data values

## Version 1 09/26/2023

In-Press Preview

## Version 2 11/08/2023

Electronic publication
