# Supplementary material for: Virus-specific TRM cells of both donor and recipient origin reside in human kidney transplants
Source: JCI Insight. 2023 Nov 8;8(21):e172681. doi: 10.1172/jci.insight.172681 (PMC10721264; doi:10.1172/jci.insight.172681)
Supplement: Supplemental data [file jciinsight-8-172681-s105.pdf]

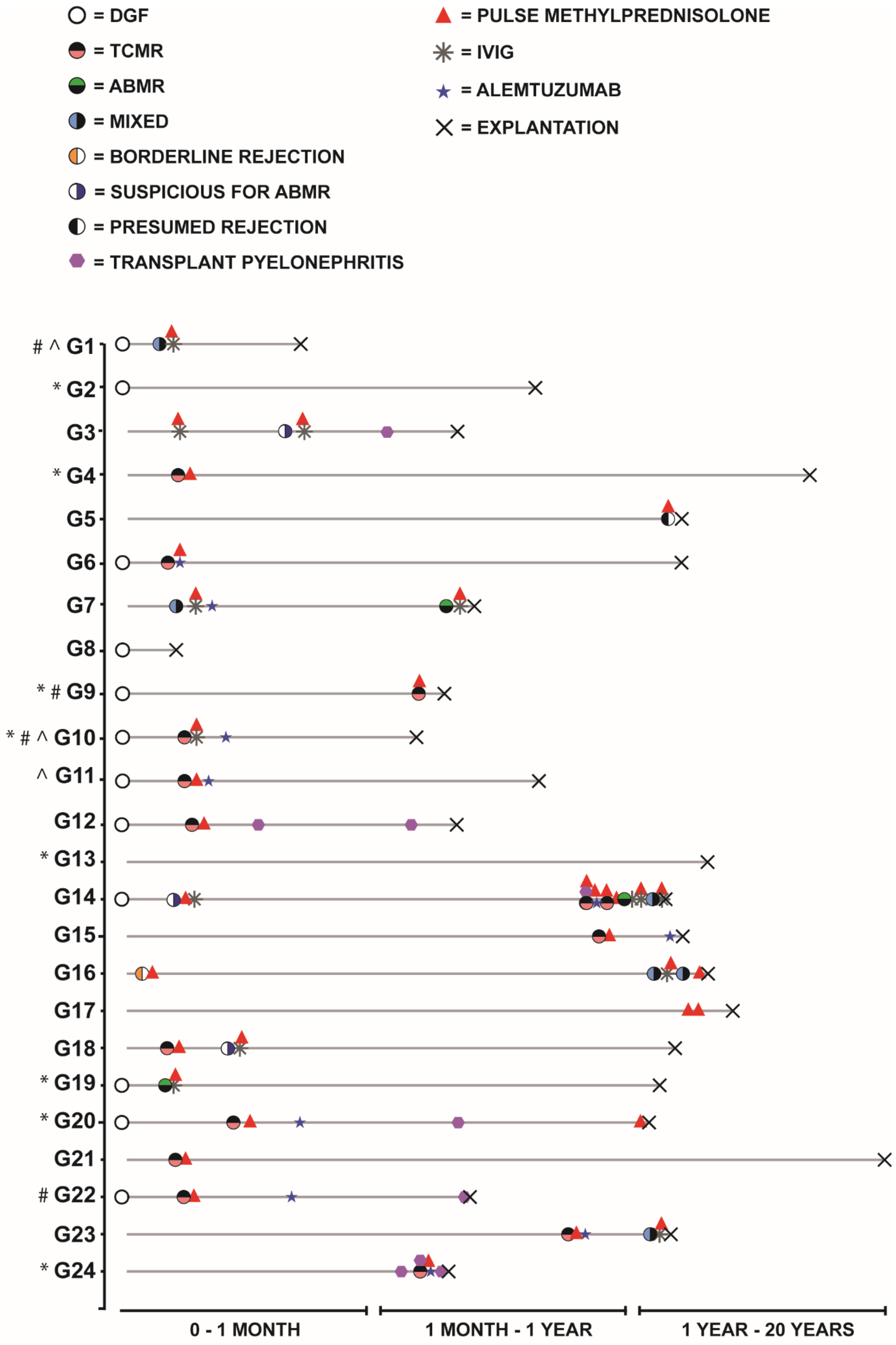
= DGF
= TCMR
= ABMR
= MIXED
= BORDERLINE REJECTION
= SUSPICIOUS FOR ABMR
= PRESUMED REJECTION
= TRANSPLANT PYELONEPHRITIS
= PULSE METHYLPREDNISOLONE
= IVIG
= ALEMTUZUMAB
= EXPLANTATION
^ G1
* G2
G3
* G4
G5
G6
G7
G8
* # G9
* # ^ G10
^ G11
G12
* G13
G14
G15
G16
G17
G18
* G19
* G20
G21
G22
G23
* G24
0 - 1 MONTH
1 MONTH - 1 YEAR
1 YEAR - 20 YEARS

Figure S1 Clinical timeline of included kidney transplant nephrectomies (n=24)
Visualization of major clinical events from transplantation until kidney transplant explantation. *G1-G24 = Study sample IDs; DGF = delayed graft function; PNF = primary non function; TCMR = T cell-mediated rejection; ABMR = antibody-mediated rejection; MIXED = mixed-type rejection; suspicious for ABMR = histomorphological signs of aABMR, but no donor-specific anti-HLA antibodies were detected and immunohistochemistry was negative for C4d; presumed rejection = presumed clinical rejection for which antirejection treatment was started but no biopsy was performed;*
** = samples used in virus dextramer experiments*
# = samples used in single-cell sequencing experiments
^ = *peripheral blood samples used in flow cytometry experiments*

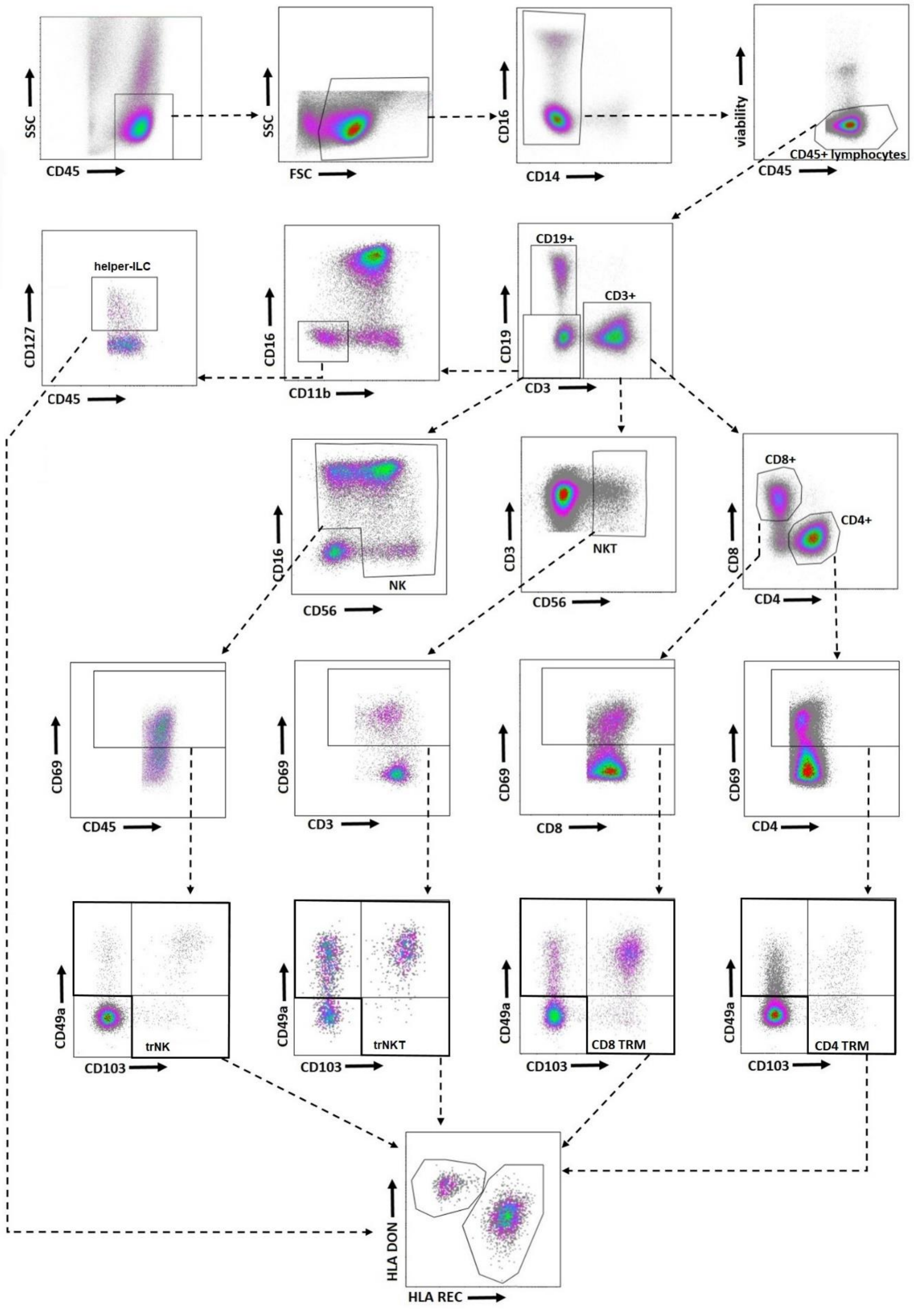
SSC
CD45
FSC
CD16
CD14
viability
CD45+ lymphocytes
helper-ILC
CD127
CD11b
CD19+
CD3+
CD19
CD3
CD56
NK
NKT
CD8+
CD4+
CD8
CD4
CD69
CD49a
CD103
trNK
trNKT
CD8 TRM
CD4 TRM
HLA DON
HLA REC

Figure S2 Gating strategy for donor and recipient TRLs

Flow cytometric gating strategy for donor and recipient CD4 T_{RM}, CD8 T_{RM}, trNK, trNKT and helper ILC. Helper ILC are lineage– (CD14-CD3-CD19-CD16-CD11b-) and CD127+. Residency of other TRL populations is defined by the expression of CD69+ plus CD103+ and/or CD49a+. Antibodies against mismatched HLA antigens between donor and recipient were used to define the origin of each TRL population.

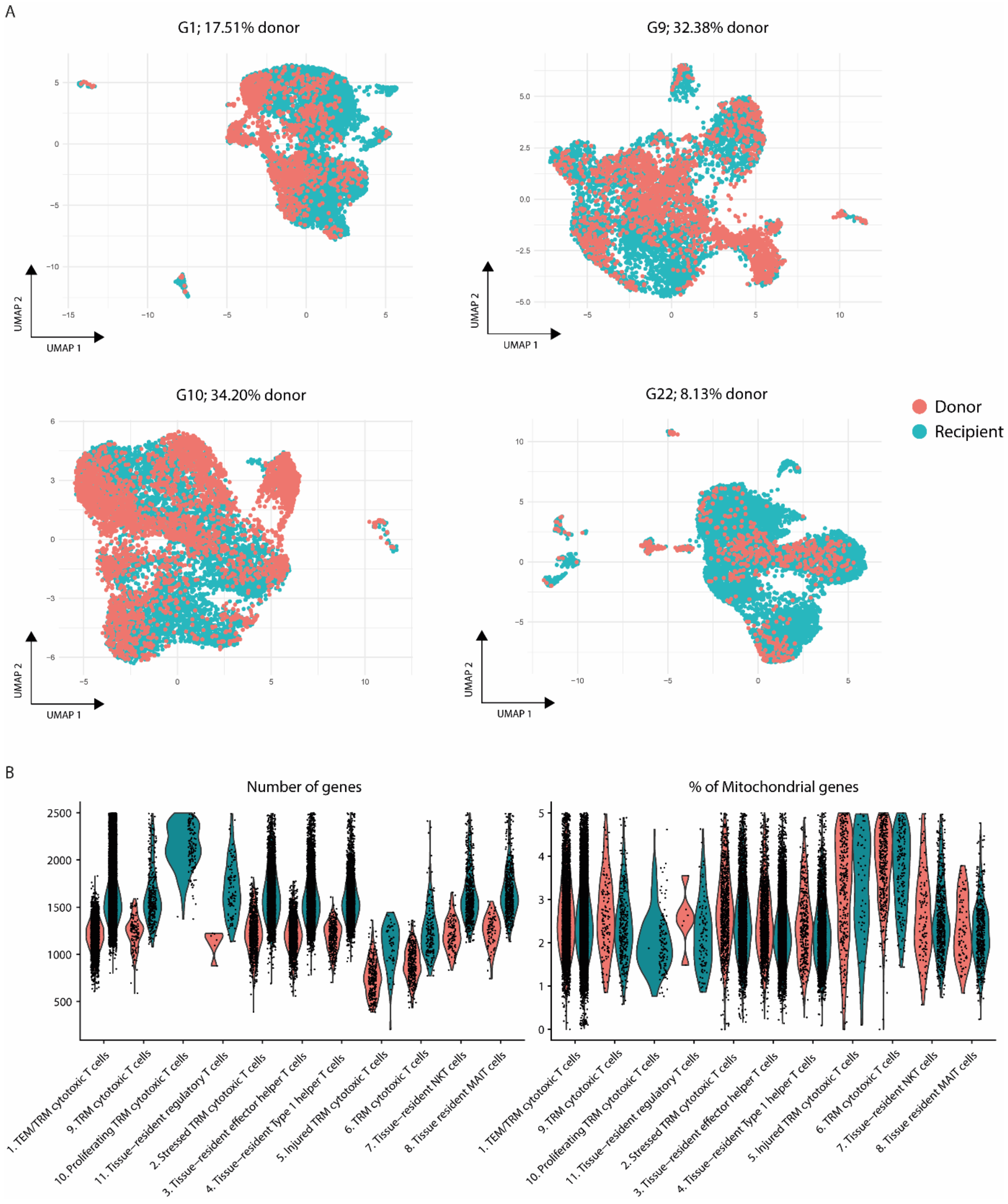

Figure S3 Single-cell RNA sequencing data optimization

(A) Demuxlet was used to determine the donor or recipient origin of cells in single-cell sequencing analysis. Donor/recipient origin of cells is shown for each of the 4 samples (study sample ID's: G1, G9, G10, G22). **(B)** Cells with <250 genes or >2500 genes expressed (left) and cells with >5% mitochondrial genes (right) were removed from analysis. Violin plots show cleaned data cell density labelled by donor/recipient origin.

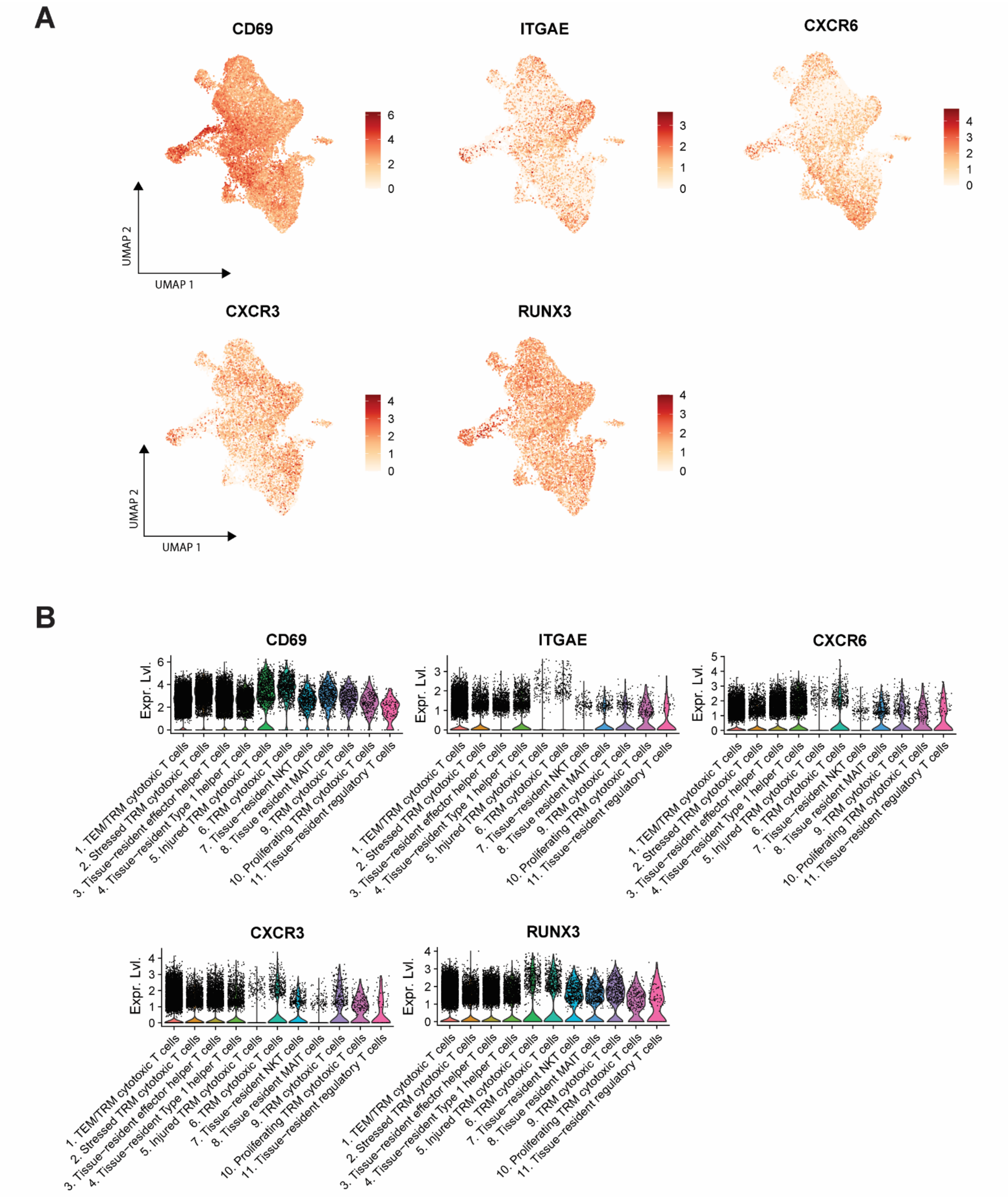

Figure S4 Tissue-residency confirmation of single-cell RNA sequencing data

(A) UMAPs show the expression of tissue-residency genes *CD69*, *ITGAE*, *CXCR6*, *CXCR3*, and *RUNX3*. **(B)** Violin plots show the expression of these genes per cell cluster.

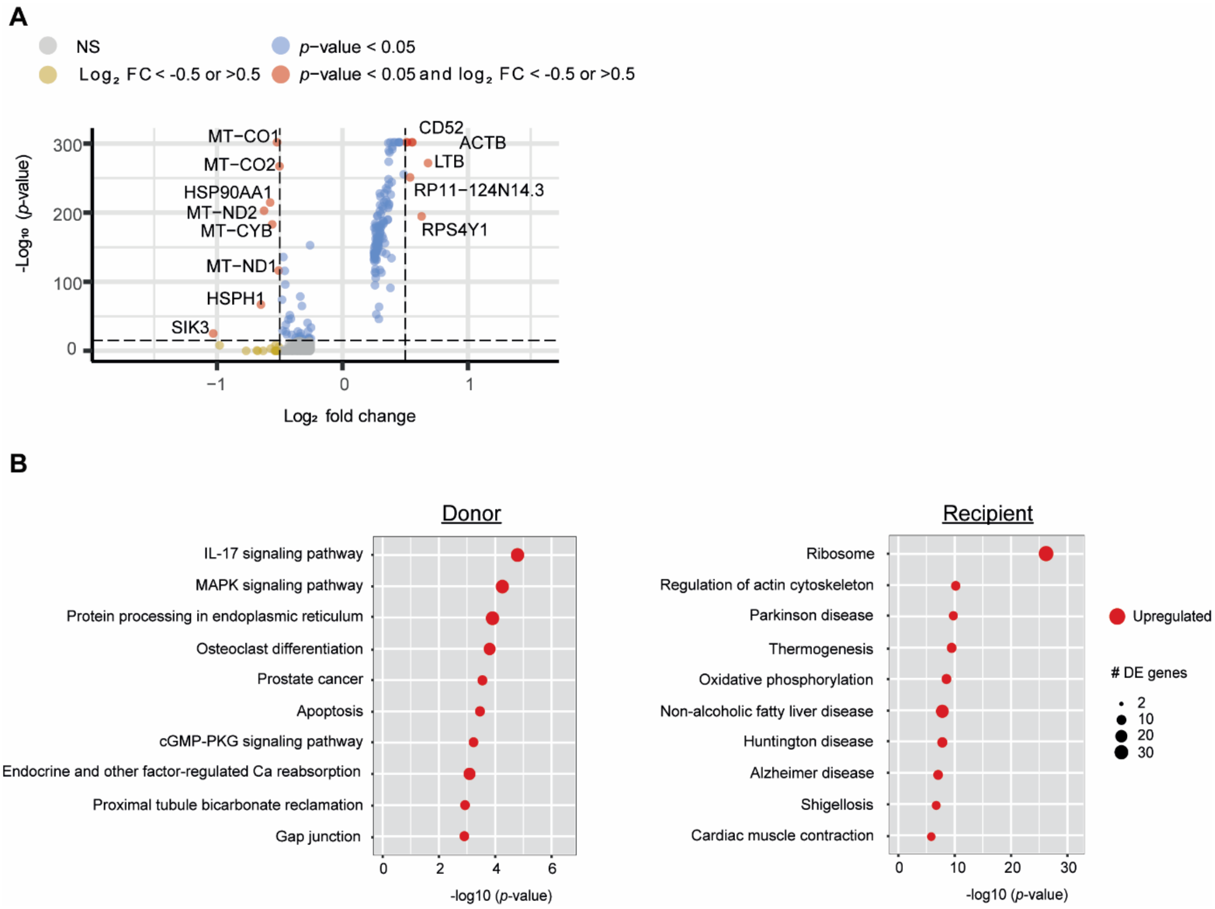

Figure S5 DEG and pathway analysis of donor and recipient T_{RM} cells.

DEG and KEGG pathway analysis was performed on the single-cell RNA sequencing data. **(A)** Volcano plot showing the significant DEG in recipient T_{RM} cells on the right and donor T_{RM} cells on the left. **(B)** Top 10 of significantly upregulated pathways in donor compared to recipient T_{RM} cells (left) and in recipient compared to donor T_{RM} cells (right).

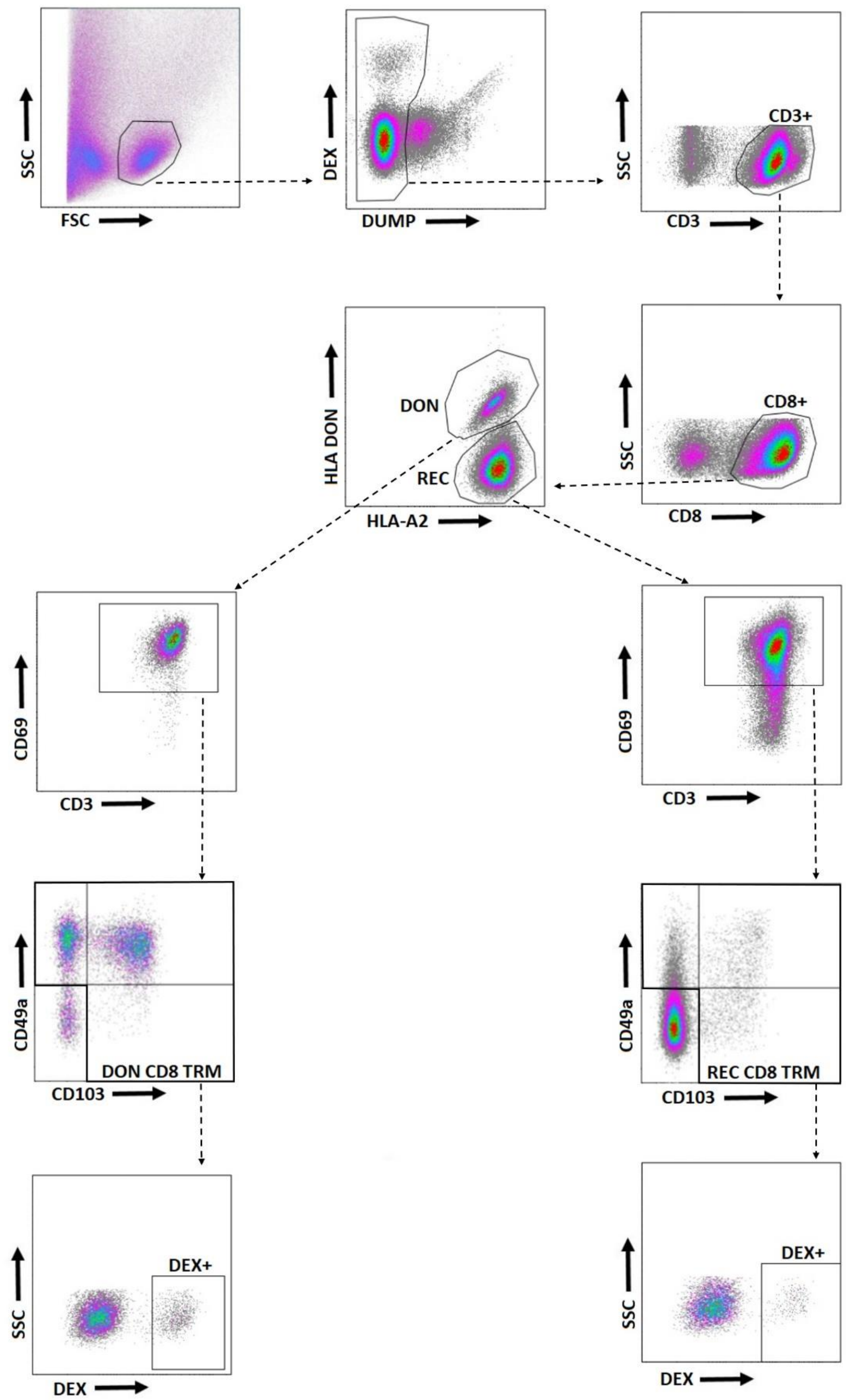

Figure S6 Gating strategy virus dextramer experiments
Flow cytometric analysis of donor and recipient CD8 T_{RM} cells to define their virus specificity against common viral antigens. HLA-A*02:01 dextramers loaded with EBV, BKV, CMV, and Influenza A were used. Lymphocytes were defined with forward and sideward scatter. CD4+ and dead cells (DUMP) were excluded. Next, donor and recipient CD8 T_{RM} cells were selected and dextramer positivity was determined.

Table S1: Baseline characteristics of kidney transplant nephrectomies (n=24)

Variables		Missing (n)
**Recipient characteristics**		
Gender (female), *n* (%)	9 (37.5%)	0
Recipient age at nephrectomy (years), median (IQR)	63.0 (46.8-68.0)	0
Primary kidney disease, n (%)		0
Hypertensive nephropathy	4 (16.7%)	
Diabetic nephropathy	8 (33.3%)	
Glomerulonephritis	3 (12.5%)	
Polycystic kidney disease	2 (8.3%)	
Reflux nephropathy	1 (4.2%)	
Other	5 (20.8%)	
Unknown	1 (4.2%)	
Transplant number (first), *n* (%)	20 (83.3%)	0
CMV positive (pretransplantation), *n* (%)	21 (87.5%)	0
EBV positive (pretransplantation), *n* (%)	21 (87.5%)	0
**Transplant characteristics**		
Donor age (years), median (IQR)	63.0 (54.0-71.0)	1
Donor type (living), *n* (%)	6 (25.0%)	0
HLA mismatches broad (HLA-A,-B and -DR), median (IQR)	3.0 (3.0-5.0)	0
CMV positive donor, *n* (%)	16 (66.7%)	0
EBV positive donor, *n* (%)	22 (100%)	2
Time to explantation (days), median (IQR)	778.5(135.0-1530.3)	0
Reason for removal, *n* (%)		0
Ongoing rejection	16 (66.7%)	
To create space for new a kidney transplant	3 (12.5%)	
Other	5 (20.8%)	

**IQR = interquartile range*

Table S2 Conclusion of routine histo-pathological examination of kidney transplant nephrectomies (n=24)

Study ID	Histo-pathological assessment*
**G1**	TCMR IIB and aABMR
**G2**	cTCMR
**G3**	aTCMR III
**G4**	ESKD
**G5**	caTCMR and caABMR, pyelonephritis and diabetic nephropathy
**G6**	cTCMR
**G7**	aABMR, C4d+
**G8**	renal vein thrombosis
**G9**	aTCMR III
**G10**	artery and renal vein thrombosis
**G11**	aTCMR II and caABMR, C4d+
**G12**	aTCMR III and aABMR, C4d+
**G13**	caTCMR and pyelonephritis
**G14**	cTCMR and aABMR, C4d+
**G15**	aTCMR III and diabetic nephropathy
**G16**	caABMR and caTCMR
**G17**	ESKD
**G18**	ESKD and artery thrombosis
**G19**	ESKD
**G20**	caABMR and caTCMR
**G21**	caTCMR
**G22**	pyelonephritis
**G23**	cTMA
**G24**	pyelonephritis, CMV infection (VIB and CMV+ cells detected by IHC), and cTCMR

**Assessed in accordance with the Banff 2019 classification*

***aABMR= acute antibody-mediated rejection, caABMR= chronic active antibody-mediated rejection TCMR= T cell-mediated rejection, aTCMR= acute T cell-mediated rejection, cTCMR= chronic T cell-mediated rejection, caTCMR= chronic active T cell-mediated rejection, cTMA= chronic thrombotic microangiopathy, ESKD= end stage kidney disease; VIB= viral inclusion bodies; IHC= immunohistochemistry*

Table S3 Definition of TRLs

Tissue-resident lymphocytes	Flow cytometry gating criteria
**CD4 T$_{RM}$**	Living CD45+CD3+CD4+CD69+CD103+CD49a-
	Living CD45+CD3+CD4+CD69+CD103+CD49a+
	Living CD45+CD3+CD4+CD69+CD103-CD49a+
**CD8 T$_{RM}$**	Living CD45+CD3+CD8+CD69+CD103+CD49a-
	Living CD45+CD3+CD8+CD69+CD103+CD49a+
	Living CD45+CD3+CD8+CD69+CD103-CD49a+
**trNK**	Living CD45+CD3-CD19-CD56+CD16-CD69+CD103+CD49a-
	Living CD45+CD3-CD19-CD56+CD16-CD69+CD103+CD49a+
	Living CD45+CD3-CD19-CD56+CD16-CD69+CD103-CD49a+
	Living CD45+CD3-CD19-CD56+CD16+CD69+CD103+CD49a-
	Living CD45+CD3-CD19-CD56+CD16+CD69+CD103+CD49a+
	Living CD45+CD3-CD19-CD56+CD16+CD69+CD103-CD49a+
	Living CD45+CD3-CD19-CD56-CD16+CD69+CD103+CD49a-
	Living CD45+CD3-CD19-CD56-CD16+CD69+CD103+CD49a+
	Living CD45+CD3-CD19-CD56-CD16+CD69+CD103-CD49a+
**trNKT**	Living CD45+CD3+CD56+CD69+CD103+CD49a-
	Living CD45+CD3+CD56+CD69+CD103+CD49a+
	Living CD45+CD3+CD56+CD69+CD103-CD49a+
**Helper ILC**	Living CD45+CD3-CD19-CD11b-CD16-CD127+

Table S4: Fluorescent Antibodies Used in Flow Cytometry Experiments

Target	Clone	Fluorochrome	Supplier*	Panel**
CD4	RPA-T4	PerCP-Cy5	BD Biosciences	1, 2
CD69	FN50	APC	Biolegend	1, 2, 3
CD3	UCHT1	AF700	Biolegend	1, 2
CD45	2D1	BV510	Biolegend	1
CD8	RPA-T8	BV570	Biolegend	1
CD11b	ICRF44	BV605	Biolegend	1
CD16	3G8	BV650	Biolegend	1
CD14	63D3	BV711	Biolegend	1
CD45RO	UCHL1	BV750	Biolegend	1
CD127	A019D5	BV785	Biolegend	1
CD49a	TS2/7	PE	Biolegend	1, 3
CD56	HCD56	PE/Dazzle	Biolegend	1
CD19	HIB19	PE-Cy5	Biolegend	1
CD103	Ber-ACT8	PE-Cy7	Biolegend	1, 3
Live/dead (Fixable-viability 780)	-	APC-Cy7	BD Biosciences	1
HLA-A2	BB7.2	BV421	BD Biosciences	1, 2
HLA-A3	GAP.A3	BV421	BD Biosciences	1, 2
HLA-B7	BB7.1	BV421	BD Biosciences	1, 2
HLA-B44/B45	-	AF488	LUMC; Abcam	1, 2
HLA-A1/A9	-	AF488	LUMC; Abcam	1, 2
HLA-A32/A23/A25/B49/B38/B58	-	AF488	LUMC; Abcam	1, 2
HLA-B51/B35	-	AF488	LUMC; Abcam	1, 2
HLA-B72/B62/B46	-	AF488	LUMC; Abcam	1, 2
Bw4	-	AF488	LUMC; Abcam	1, 2
Live/dead (7-AAD)	-	PerCP	BD Biosciences	2, 3
CD8	SK1	APC-Cy7	Biolegend	2, 3
CD103	Ber-ACT8	BV711	Biolegend	2
CD49a	TS2/7	PE-Cy7	Biolegend	2

CD3	OKT3	BV510	Biolegend	3
CD4	RPA-T4	BV421	Biolegend	3
Isotype, mouse IgG1 κ	MOPC-21	APC	Biolegend	1
Isotype, mouse IgG1 κ	MOPC-21	PE-Cy7	Biolegend	1
Isotype, mouse IgG1 κ	MOPC-21	PE	Biolegend	1
Isotype, mouse IgG1 κ	MOPC-21	BV605	Biolegend	1
Isotype, mouse IgG1 κ	MOPC-21	BV785	Biolegend	1
Isotype, mouse IgG1 κ	MOPC-21	AF488	Biolegend	1
Isotype, mouse IgG1 κ	MOPC-21	BV421	Biolegend	1

**BD Biosciences (Franklin Lakes, NJ, USA); Biolegend (San Diego, CA, USA), LUMC (Leiden, the Netherlands); Abcam (Cambridge, UK) ** Panel 1=Staining of donor and recipient tissue resident lymphocytes; Panel 2=Staining of CD8 T_{RM} cells with virus dextramers; Panel 3=FACSort of T_{RM} cells for single cell sequencing*

Table S5: Sorted cell populations for single-cell sequencing

Cell populations
Living CD3+ CD4+ CD69+ CD103+ CD49a+
Living CD3+ CD4+ CD69+ CD103- CD49a+
Living CD3+ CD4+ CD69+ CD103+ CD49a-
Living CD3+ CD8+ CD69+ CD103+ CD49a+
Living CD3+ CD8+ CD69+ CD103- CD49a+
Living CD3+ CD8+ CD69+ CD103+ CD49a-

Table S6 → Excel file

Table S7 Single cell TCR sequencing recovery

	% TCR recovery	min. 1 alpha chain	min. 1 beta chain	min 1 alpha + 1 beta chain
**DONOR**	73.02%	53.27%	68.04%	48.30%
**RECIPIENT**	84.55%	71.58%	79.65%	66.69%
**TOTAL**	81.79%	67.21%	76.88%	62.29%

Table S8 Distribution of clone sizes across donor and recipient T_{RM} cells

Cell Cluster ID	Clone size	Number of cells per clone size	Number of total cells in cell cluster	Proportion of clone size per cell cluster
DONOR	Hyperexpanded (100 < X <= 500)	438	8443	0.0519
RECIPIENT	Hyperexpanded (100 < X <= 500)	2684	26866	0.0999
DONOR	Large (20 < X <= 100)	1349	8443	0.1598
RECIPIENT	Large (20 < X <= 100)	4513	26866	0.1680
DONOR	Medium (5 < X <= 20)	1287	8443	0.1524
RECIPIENT	Medium (5 < X <= 20)	4240	26866	0.1578
DONOR	Small (1 < X <= 5)	1291	8443	0.1529
RECIPIENT	Small (1 < X <= 5)	4671	26866	0.1739
DONOR	Single (0 < X <= 1)	1800	8443	0.2132
RECIPIENT	Single (0 < X <= 1)	6606	26866	0.2459

Table S9 Distribution of clone sizes across cell clusters

Cell cluster name (cluster number)	Clone size	Number of cells per clone size	Number of total cells in cell cluster	Proportion of clone size per cell cluster
TEM/TRM cytotoxic T cells (1)	Hyperexpanded (100 < X <= 500)	2170	13759	0.1577
TRM cytotoxic T cells (9)	Hyperexpanded (100 < X <= 500)	28	368	0.0761
Proliferating TRM cytotoxic T cells (10)	Hyperexpanded (100 < X <= 500)	32	192	0.1667
Stressed TRM cytotoxic T cells (2)	Hyperexpanded (100 < X <= 500)	350	3373	0.1038
Tissue-resident effector helper T cells (3)	Hyperexpanded (100 < X <= 500)	71	6475	0.0110
Tissue-resident Type 1 helper T cells (4)	Hyperexpanded (100 < X <= 500)	372	2779	0.1339
Injured TRM cytotoxic T cells (5)	Hyperexpanded (100 < X <= 500)	21	230	0.0913
TRM cytotoxic T cells (6)	Hyperexpanded (100 < X <= 500)	50	612	0.0817
Tissue-resident NKT cells (7)	Hyperexpanded (100 < X <= 500)	22	511	0.0431
Tissue-resident MAIT cells (8)	Hyperexpanded (100 < X <= 500)	6	471	0.0127
TEM/TRM cytotoxic T cells (1)	Large (20 < X <= 100)	2710	13759	0.1970
TRM cytotoxic T cells (9)	Large (20 < X <= 100)	76	368	0.2065
Proliferating TRM cytotoxic T cells (10)	Large (20 < X <= 100)	59	192	0.3073
Tissue-resident regulatory T cells (11)	Large (20 < X <= 100)	3	109	0.0275
Stressed TRM cytotoxic T cells (2)	Large (20 < X <= 100)	821	3373	0.2434
Tissue-resident effector helper T cells (3)	Large (20 < X <= 100)	983	6475	0.1518
Tissue-resident Type 1 helper T cells (4)	Large (20 < X <= 100)	801	2779	0.2882
Injured TRM cytotoxic T cells (5)	Large (20 < X <= 100)	27	230	0.1174
TRM cytotoxic T cells (6)	Large (20 < X <= 100)	128	612	0.2092
Tissue-resident NKT cells (7)	Large (20 < X <= 100)	197	511	0.3855
Tissue-resident MAIT cells (8)	Large (20 < X <= 100)	57	471	0.1210
TEM/TRM cytotoxic T cells (1)	Medium (5 < X <= 20)	2739	13759	0.1991
TRM cytotoxic T cells (9)	Medium (5 < X <= 20)	86	368	0.2337
Proliferating TRM cytotoxic T cells (10)	Medium (5 < X <= 20)	34	192	0.1771
Tissue-resident regulatory T cells (11)	Medium (5 < X <= 20)	2	109	0.0183
Stressed TRM cytotoxic T cells (2)	Medium (5 < X <= 20)	721	3373	0.2138
Tissue-resident effector helper T cells (3)	Medium (5 < X <= 20)	986	6475	0.1523
Tissue-resident Type 1 helper T cells (4)	Medium (5 < X <= 20)	641	2779	0.2307
Injured TRM cytotoxic T cells (5)	Medium (5 < X <= 20)	34	230	0.1478
TRM cytotoxic T cells (6)	Medium (5 < X <= 20)	114	612	0.1863
Tissue-resident NKT cells (7)	Medium (5 < X <= 20)	83	511	0.1624
Tissue-resident MAIT cells (8)	Medium (5 < X <= 20)	87	471	0.1847
TEM/TRM cytotoxic T cells (1)	Small (1 < X <= 5)	2756	13759	0.2003
TRM cytotoxic T cells (9)	Small (1 < X <= 5)	72	368	0.1957
Proliferating TRM cytotoxic T cells (10)	Small (1 < X <= 5)	36	192	0.1875
Tissue-resident regulatory T cells (11)	Small (1 < X <= 5)	18	109	0.1651
Stressed TRM cytotoxic T cells (2)	Small (1 < X <= 5)	705	3373	0.2090
Tissue-resident effector helper T cells (3)	Small (1 < X <= 5)	1465	6475	0.2263
Tissue-resident Type 1 helper T cells (4)	Small (1 < X <= 5)	516	2779	0.1857
Injured TRM cytotoxic T cells (5)	Small (1 < X <= 5)	52	230	0.2261
TRM cytotoxic T cells (6)	Small (1 < X <= 5)	119	612	0.1944

Tissue-resident NKT cells (7)	Small (1 < X <= 5)	94	511	0.1840
Tissue-resident MAIT cells (8)	Small (1 < X <= 5)	129	471	0.2739
TEM/TRM cytotoxic T cells (1)	Single (0 < X <= 1)	3384	13759	0.2459
TRM cytotoxic T cells (9)	Single (0 < X <= 1)	106	368	0.2880
Proliferating TRM cytotoxic T cells (10)	Single (0 < X <= 1)	31	192	0.1615
Tissue-resident regulatory T cells (11)	Single (0 < X <= 1)	86	109	0.7890
Stressed TRM cytotoxic T cells (2)	Single (0 < X <= 1)	776	3373	0.2301
Tissue-resident effector helper T cells (3)	Single (0 < X <= 1)	2970	6475	0.4587
Tissue-resident Type 1 helper T cells (4)	Single (0 < X <= 1)	449	2779	0.1616
Injured TRM cytotoxic T cells (5)	Single (0 < X <= 1)	96	230	0.4174
TRM cytotoxic T cells (6)	Single (0 < X <= 1)	201	612	0.3284
Tissue-resident NKT cells (7)	Single (0 < X <= 1)	115	511	0.2250
Tissue-resident MAIT cells (8)	Single (0 < X <= 1)	192	471	0.4076

Table S10 → excel

Table S11 → excel

Table S12 → excel

Table S13: Virus Dextramers

Virus	Antigen	Peptide	Allele	Fluorochrome	Supplier*
EBV	BMLF1	GLCTLVAML	HLA-A*02:01	PE	Immudex
BKV	VP1	LLMWEAVTV	HLA-A*02:01	PE	Immudex
CMV	pp65	NLVPMVATV	HLA-A*02:01	PE	Immudex
Influenza A	MP	GILGFVFTL	HLA-A*02:01	PE	Immudex
Negative control	-	-	HLA-A*02:01	PE	Immudex

* *Immudex (Virum, Denmark)*

Table S14 → excel
